# Genome-Wide Identification, Classification, and Expression Analysis of *Amino Acid Transporter* Gene Family in *Glycine Max*

**DOI:** 10.3389/fpls.2016.00515

**Published:** 2016-04-20

**Authors:** Lin Cheng, Hong-Yu Yuan, Ren Ren, Shi-Qi Zhao, Ya-Peng Han, Qi-Ying Zhou, Dan-Xia Ke, Ying-Xiang Wang, Lei Wang

**Affiliations:** ^1^Bioinformatics Laboratory, College of Life Sciences, Xinyang Normal UniversityXinyang, China; ^2^Institute for Conservation and Utilization of Agro-Bioresources in Dabie MountainsXinyang, China; ^3^State Key Laboratory of Genetic Engineering and Institute of Genetics, Institute of Plant Biology, School of Life Sciences, Fudan UniversityShanghai, China

**Keywords:** soybean (*Glycine max* L.), *AAT* gene family, genome-wide analysis, phylogeny, gene structure, expression pattern

## Abstract

Amino acid transporters (AATs) play important roles in transporting amino acid across cellular membranes and are essential for plant growth and development. To date, the *AAT* gene family in soybean (*Glycine max* L.) has not been characterized. In this study, we identified 189 *AAT* genes from the entire soybean genomic sequence, and classified them into 12 distinct subfamilies based upon their sequence composition and phylogenetic positions. To further investigate the functions of these genes, we analyzed the chromosome distributions, gene structures, duplication patterns, phylogenetic tree, tissue expression patterns of the 189 *AAT* genes in soybean. We found that a large number of *AAT* genes in soybean were expanded via gene duplication, 46 and 36 *GmAAT* genes were WGD/segmental and tandemly duplicated, respectively. Further comprehensive analyses of the expression profiles of *GmAAT* genes in various stages of vegetative and reproductive development showed that soybean *AAT* genes exhibited preferential or distinct expression patterns among different tissues. Overall, our study provides a framework for further analysis of the biological functions of *AAT* genes in either soybean or other crops.

## Introduction

Amino acids are the currency of nitrogen exchange in plants (Ortiz-Lopez et al., 2000) and are the second most abundant class of organic compounds found in the phloem sap after sucrose (Rentsch et al., 1998). Amino acid transporters (AATs) function in long distance amino acid transport and are essential participants in the resource allocation processes that support plant growth, development, and responses to pathogen and abiotic stresses (Tegeder, 2012). The *AAT* genes are distinguished by the presence of PF01490 (Aa_trans) and PF00324 (Aa_permease) domains. More than 63 distinct *AAT* genes have been identified in *Arabidopsis* by means of heterologous expression systems and database screening with known transporters (Rentsch et al., 2007). Emerging evidence also identified 85 *AAT* genes in rice (Zhao et al., 2012), suggesting that this gene family is widely existed in higher plants.

The *AAT* gene family in plants includes the amino acid/auxin permease (AAAP) family and the amino acid-polyamine-choline (APC) transporter family, which belong to the APC transporter superfamily. The APC family is further grouped into cationic amino acid transporters (CATs), amino acid/choline transporters (ACTs) and polyamine H^+^-symporters (PHSs) subfamilies (Tegeder, 2012). The AAAP family comprises c-aminobutyric acid transporters (GATs), proline transporters (ProTs), Amino acid permeases (AAPs), lysine and histidine transporters (LHTs), auxin transporters (AUXs), and aromatic and neutral amino acid transporters (ANTs) (Saier et al., 2009; Hunt et al., 2010; Okumoto and Pilot, 2011).

Many AATs have been functionally studied in *Arabidopsis* and rice (*Oryza sativa*) with eight members of *AtAAP1-AtAAP8* in *Arabidopsis* and nineteen members of *OsAAP1-OsAAP19* in rice, respectively (Okumoto et al., 2002; Couturier et al., 2010). In particular, detailed characterizations of *AtAAP1*-*AtAAP6* and *AtAAP8* have been carried out using heterologous expression systems, and these genes preferentially transport neutral and charged amino acids with varying specificities and affinities (Su et al., 2004). *AtAAP1* is produced in *Arabidopsis* embryos, and mediated uptake of amino acids by the embryo is importance for storage protein synthesis and seed yield (Lee et al., 2007; Sanders et al., 2009). *AtAAP2* functions in xylem-tophloem transfor (Zhang et al., 2010). *AtAAP6* regulates phloem amino acid composition, thereby affecting interactions with aphids (Hunt et al., 2010). *AtAAP8* plays a crucial role in the uptake of amino acids into the endosperm and in supplying the developing embryo with amino acids during early embryogenesis (Schmidt et al., 2007). The functions of *AAP* genes have also been studies in other species such as *Vicia faba* (Miranda et al., 2001), *Solanum tuberosum* (Koch et al., 2003), and *Populus trichocarpa* (Couturier et al., 2010). For example, *VfAAP1* is highly expressed in the cotyledons of *Vicia faba* at early developmental stages and shows moderate expression in other sink tissues (Miranda et al., 2001). Moreover, *VfAAP3* is expressed most abundantly in maternal tissues of roots, stems, gynoecia, pods and seed coats at different developmental stages. *VfAAP4* transcripts was undetectable by northern hybridization (Miranda et al., 2001).

Many *AAT* genes are known function in mitigating water stress conditions in plants especially by facilitating the transport of stress-related compounds and compatible solutes, such as proline, betaine, GABA, and a variety of carbohydrates (Serrano, 1996). For example, *ProT* encodes an AAT protein and is a high affinity proline specific transporter (ProT) that rapidly distributes the proline under water stress conditions (Lehmann et al., 2010). Proline transporters including *OsProT, AtProT1*, and *LeProT1* were detected using a transport assay in rice, *Arabidopsis*, and tomato, respectively (Schwacke et al., 1999; Grallath et al., 2005; Lehmann et al., 2011). In tomato, *LeProT1* was specifically expressed in both mature and germinating pollen, and transports proline and γ-amino butyric acid with low affinity and glycine betaine with high affinity (Schwacke et al., 1999). In *Arabidopsis, AtProT* was highly expressed in tissues with elevated proline content such as in pollen and the epidermides of leaves (Lehmann et al., 2011). *AtProT2* was shown to facilitate uptake of L- and D-proline as well as [^14^C] glycine betaine in plants, and these functions indicate in importing compatible solutes into the root (Lehmann et al., 2011). *AAT* genes also function in auxin transport-dependent plant growth and development (Swarup et al., 2008). *AtAUX1* is an auxin influx carrier in roots and facilitates gravitropism and lateral root formation (Marchant et al., 1999, 2002). *AtBAT1* is similar to a yeast GABA transporter (UGA4) and has been isolated as a bi-directional amino acid transporter (Dündar and Bush, 2009). Recently, *AtBAT1* was characterized to be expressed in the mitochondrial membrane and mediates the transport of GABA from the cytosol into mitochondria (Michaeli et al., 2011).

Soybean (*Glycine max* L.) is a legume plant belonging to the Papilionoideae family and is a rich source of protein, oil and plant natural products such as isoflavonoids. The soybean genome contains 56,044 protein coding loci located on 20 different chromosomes. Soybean has undergone two whole genome duplication (WGD) events approximately 59 and 13 million years ago, leading to the presence ~85% paralogs in its genome (Shoemaker et al., 1996; Schmutz et al., 2010; Lehmann et al., 2011). Although *AAT* genes have been well-characterized in *Arapidopsis*, rice, and other plants, there have been no such studies in soybean. The complete sequence of soybean genome has enabled gene prediction tools and annotation to become publicly available. In the current study, we found that soybean has 189 *AAT* genes, which is larger than that in *Arabidopsis* and rice. Phylogenetic analysis divided 189 *GmAATs* into 12 clusters. Identification of intron/exon structural patterns and conserver motifs among 189 AAT members showed a high consistence between gene organization and protein structure. Expression patterns of the 189 genes in different tissues suggest that they may have functional conservation and divergence. We expect that our results provide a framework for the validation of the soybean *AAT* genes and broaden our understandings of the roles of *AATs* in plants.

## Materials and methods

### Database screening and sequence collection

The genome sequence of soybean was obtained from Phytozome (http://phytozome.jgi.doe.gov/pz/portal.html). To find putative AAT members in the soybean genome, several approaches were employed. First, the Hidden Markov Model (HMM) profile of the AAT domain (PF01490 and PF00324) was obtained from the Pfam website (http://pfam.xfam.org/), which was employed as a query to identify all possible *AAT* genes in soybean using HMMER v3.0 software. Second, we downloaded protein sequences of putative AAT members in soybean from Search Interpro (http://www.ebi.ac.uk/interpro/ISearch?query = PF01490 and PF00324). After removing the redundant sequences, we submitted the remaining protein sequences to InterProScan (http://www.ebi.ac.uk/interpro/scan.html) to confirm the existence of AAT domains. After determining the *AAT* genes in the soybean genome, we acquired information about each gene including full-length cDNA accessions, coding sequence length, gene structure, and protein product characteristics. We analyzed the structure of *GmAATs* using tools on the GSDS website (http://gsds1.cbi.pku.edu.cn/index.php), predicted the putative transmembrane (TM) regions in each GmAAT protein using the TMHMM Sever v2.0 (http://www.cbs.dtu.dk/services/TMHMM/) with default settings.

### Chromosomal localization and duplication of *AAT* genes in soybean

The chromosomal positions of *GmAAT* genes were mapped in Phytozome 10.1 (http://phytozome.jgi.doe.gov/pz/portal.html#!info?alias=Org_Gmax). We used MapChart to draw and annotate the *GmAAT* genes on chromosomes. The duplication events of *GmAAT* genes were explored by Multiple Collonearity Scan toolkit (MCScanx: http://chibba.pgml.uga.edu/mcscan2/). Tandem duplications were identified as multiple members of the AAT gene family occurring within the same or neighboring intergenic regions. WGD/segmental duplications of AAT gene within the family in soybean were searched in the PGDD (http://chibba.agtec.uga.edu/duplication/).

### Analysis of Ka, Ks and calculation of the duplication event

We calculated the number of synonymous (Ks) and non-synonymous (Ka) substitutions per site of duplicated *AAT* genes using the MCScanX program (http://chibba.pgml.uga.edu/mcscan2/). The Ka and Ks were used to assess selection history (Li et al., 1981) and divergence time. For evaluating selection history, we assumed that purifying selection results in a ratio of Ka/Ks < 1 if while positive selection yields while Ka/Ks > 1 (Juretic et al., 2005). To determine divergence time between gene pairs, we assumed 6.1 × 10^−9^ substitutions per site per year. Thus, we calculated the divergence time (T) as T = Ks/ (2 × 6.1 × 10^−9^) × 10^−6^ Million years (My) (Lynch and Conery, 2000).

### Phylogenetic analysis and sequence alignment

We detected the PF01490 and PF00324 domains of *GmAAT* genes using SMART (http://smart.embl-heidelberg.de/) and Pfam (http://pfam.xfam.org/). In order to identify orthologous genes between soybean and *Arabidopsis*, we generated a Maximum Likelihood tree comprising *GmAATs* and *AtAATs*. Sequence alignments were performed using MUSCLE 3.8.31 (Edgar, 2004). Phylogenetic analyses were conducted using Maximum Likelihood (ML). RAxML v8.1.3 (Stamatakis, 2014) was employed to construct ML trees, with the GTRCAT model, gamma distribution option and 100 nonparametric bootstrap replicates. Then we used MEGA 6.0 to display the phylogenetic tree (Tamura et al., 2013). The software Multiple Em for Motif Elicitation (MEME) (http://meme-suite.org/tools/meme) was used using the following parameters: the width of a motif was between 20aa and 300aa and the number of motif was 20. We analyzed the conservation of amino acid sequences in the GmAAT subfamily using DNAMAN software and modified manually and detected conserved motifs using MEME analysis. We also identified and annotated transmembrane (TM) domains using the TMHMM 2.0 server (http://www.cbs.dtu.dk/services/TMHMM/).The numbers and positions of exons and introns were determined through the comparison of full-length cDNA sequences and the corresponding genomic DNA sequences of each *AAT* gene in soybean by using GSDS (http://gsds.cbi.pku.edu.cn/).

### Expression analysis of *AATs* in 11 tissues of soybean

To analyze expression patterns of soybean *AAT*, we used RNA-seq data as generated previously (Wang et al., 2014). These data include reads from 11 tissues of soybean including root tip (RT), hypocotyl, cotyledon, callus, shoot aptical meristem at 6, 17, and 38 day stages (SAM6d, SAM17d, SAM38d), axillary meristem (AM), inflorescences before and after the meiotic stage (IBM, IAM) and open flower (OF).

The normalized gene expression level was calculated as Reads Per Kilo-base of mRNA length per Millions of mapped reads (RPKM) by the GFOLD V1.0.7 software (Feng et al., 2012). The expression patterns analysis of *AAT* genes in these tissues were performed by R software.

## Results and discussion

### Identification of *AAT* genes in soybean genome

Initially, we identified 206 putative *AAT* genes in soybean. After screening regarding on the presence of PF01490 and PF00324 domains, we removed 17 candidates because their characteristic domains were short or incomplete. Thus, we identified a total of 189 *AATs* in soybean, which is greater than that identified in other model species, including *Arabidopsis* and rice with 63 and 85 *AATs*, respectively.

We renamed the 189 *AAT* genes in soybean according to their affinities within gene subfamilies (Table S1). Details of each gene including its nucleic acid and protein sequence are provided in Table S1. The identified soybean *AAT* genes encode peptides ranging from 133 to 742 amino acids with the isoelectric points (pI) varying from 5.06 to 9.98 and molecular weights (Mw) varying from 14.3 to 81.1 kD.

To obtain fully understand relative information in soybean *AATs*, the putative transmembrane (TM) regions were predicted by TMHMM server. The number of TM regions in most *GmAATs* ranges from 0 to 15 (Table S1), and the same subfamily have the similar TM regions, such as 10 or 11 in ProT and 10 or 12 in ACT. Among which, GmACT1 and GmAAP33 were not identified with any TM with TMHMM prediction, suggesting that it might lost its function during gene expansion. Even so, the change of TM regions in the soybean was greater than in rice.

### Chromosomal distribution and duplication analysis of *AAT* family genes in soybean

**Figure 1:**
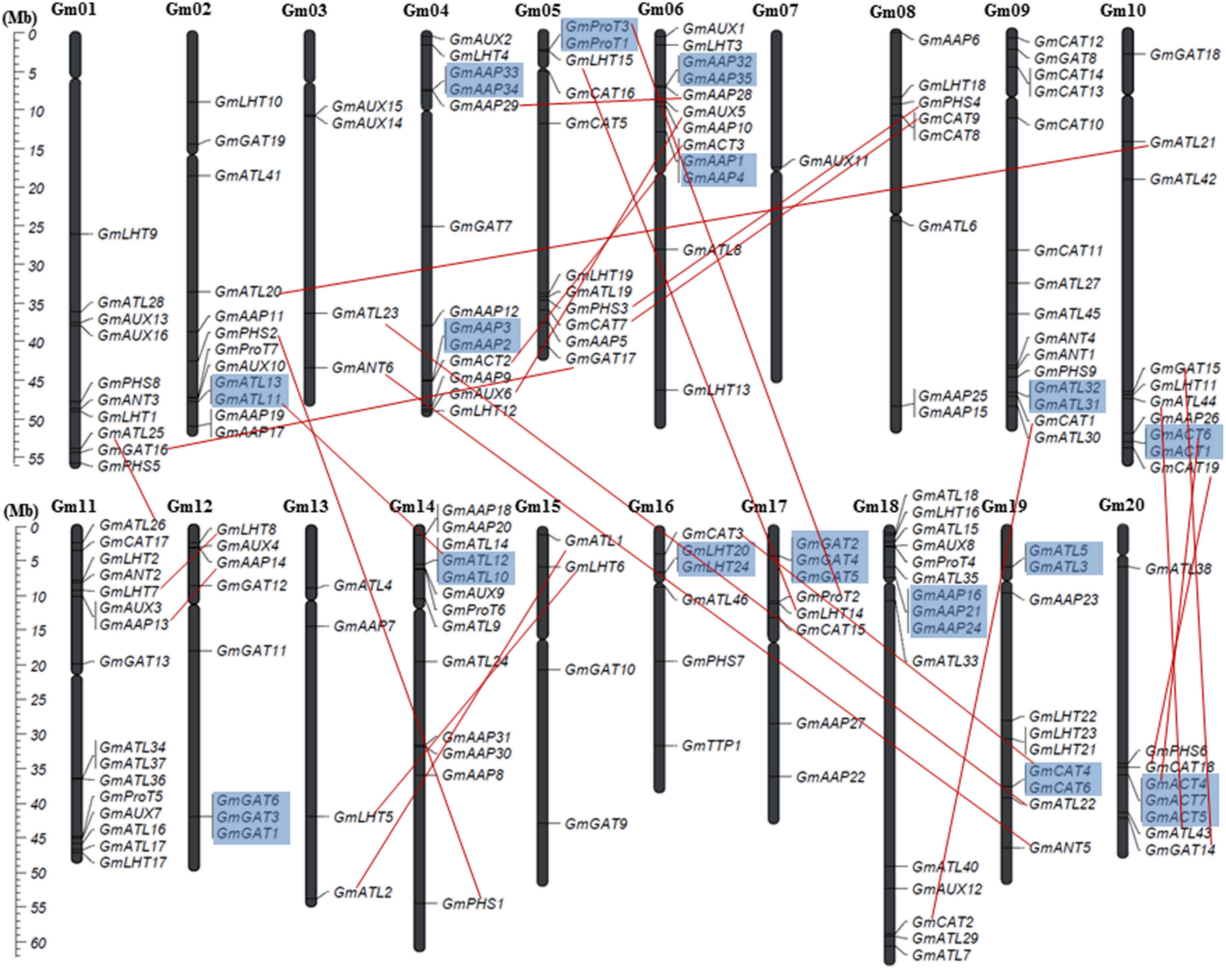
**Chromosomal localization and gene duplication events of ***GmAAT*** genes**. Respective chromosome numbers are indicated at the top of each bar. The scale on the left is in megabases (Mb). The cleavages on the chromosomes (vertical bars) indicate the positon of centromeres. 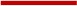 WGD/Segmentally duplicated genes 
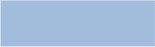
 Tandemly duplicated genes.

We mapped 188 of the 189 *AAT* genes in soybean to the 20 soybean chromosomes, and the remaining gene *GmATL39* showed affinity with yet unattributed scaffolds (Figure 1). The *AAT* genes in soybean were unevenly distributed among chromosomes, but all chromosomes possessed at least one. The largest number of *GmAAT* genes on a single chromosome was 16 on chromosome 11, while chromosomes 09, 18, and 13 possessed 15 each. In addition, 12 genes each are located on chromosome 02 and 06; 11 genes are located on chromosome 05; 10 genes each are located on chromosome 01, 10, and 19; 8 genes each are located on chromosome 08, 12, 17, and 20; 6 genes are located on chromosome 16; 4 genes each are located on chromosome 03, 13, and 15. In contrast, chromosome 07 possessed only one *GmAAT* gene. Meanwhile, duplicated segments are mainly present on chromosome 02, 04, 05, and 10, and 18 gene groups in tandem duplication are localized on 13 chromosomes (Figure 1).

Gene duplication is thought to be an important mean of gene family expansion and functional diversity evolution (Kong et al., 2007). Gene duplication is known to occur in three major ways, namely through chromosomal segmental duplication, tandem duplication and whole genome duplication (WGD). Previous studies have shown that the soybean genome has undergone two rounds of WGD that occurred approximately 59 and 13 million years ago (Mya) (Schmutz et al., 2010). However, the functions of duplicated genes are often poorly understood. Among the 189 *AAT* genes in soybean, 40.74% (77 of 189) represent duplication events, including 46 segmental duplications and 36 tandem duplications (*GmATL11, GmProT3, GmACT6, GmATL12, GmCAT4, GmACT4* are both WGD and tandem duplication genes). We were able to assign the 46 (23 pairs) WGD duplicated *GmAAT* genes to blocks based on the analysis of the MCScanX program. The 36 genes tandemly duplicated genes represented eight groups. Four groups comprised two genes each, one group comprised five genes, two groups comprised six genes, and one group comprised 11 genes. The tandemly duplicated genes are localized on 13 chromosomes; namely, two groups on chromosome 19 and one group each on chromosomes 02/04/05/06/09/10/12/14/16/ 17/18 and 20 (Figure 1). Moreover, 16 of 35 genes in GmAPC subfamily are involved in the duplication events, and the duplications probably explain the difference in the number of *APC* genes in soybean compared to *Arabidopsis*, which has only 15 total APCs (Okumoto and Pilot, 2011).

According to the ration of Ka/Ks descripted previously, the history of selection acting on coding sequence can be estimated (Li et al., 1981). Previous studies shown that the soybean genome had experienced two rounds of whole genome duplication (WGD), an ancient duplication prior to the divergence of papilionoid (58–60 Mya) and a recent *Glycine*-specific WGD event occurring approximately 13 Mya (Schmutz et al., 2010). Ks value was calculated for estimating the separation time of each paralogous gene pair (Table 1). All the Ks values ranged from 0.1 to 0.9 with two peaks at 0.1–0.2 and 0.35–0.4, which were consisted with whole genome duplication events at round 13 and 59 Mya. In addition, our divergence time analyses showed that duplications among 23 paralogous pairs occurred between 6.78 and 76.51 Mya (Table 1). We demonstrated that 20 paralogous pairs derived from the second WGD except the *GmCAT7/GmCAT9, GmPHS1/GmPHS2*, and *GmACT4/GmACT6*. It suggested the WGD duplication might be the main mechanism of *AAT* gene family expansion and functional diversity during evolution of soybean. This result consisted with some other gene families in soybean, such as *GST* supergene family (Liu et al., 2015), receptor-like protein kinase genes (Zhou et al., 2016), *HSP70* genes (Zhang et al., 2015) in soybean, or *AP2/ERF* superfamily genes and *14-3-3* family genes in *Medicago truncatula* (Chen et al., 2001; Qin et al., 2016; Shu et al., 2016).

**Table 1:** **Divergence between ***AAT*** segmentally duplicate gene pairs in soybean**.

	**Paralogous pairs**	**Ka**	**Ks**	**Ka/Ks**	**Duplication date (MY)**
1	GmCAT1-GmCAT2	0.011	0.103	0.110	8.43
2	GmCAT3-GmCAT4	0.063	0.121	0.518	9.96
3	GmCAT7-GmCAT9	0.163	0.933	0.174	76.51
4	GmCAT18-GmCAT19	0.058	0.171	0.338	13.98
5	GmPHS1-GmPHS2	0.061	0.348	0.176	28.53
6	GmPHS3-GmPHS4	0.037	0.114	0.326	9.32
7	GmACT2-GmACT3	0.007	0.121	0.061	9.95
8	GmACT4-GmACT6	0.088	0.400	0.220	32.81
9	GmATL1-GmATL2	0.010	0.102	0.099	8.38
10	GmATL11-GmATL12	0.244	0.228	1.070	18.65
11	ANT5-ANT6	0.033	0.109	0.304	8.91
12	ATL20-ATL21	0.101	0.201	0.504	16.49
13	ATL22-ATL23	0.098	0.165	0.596	13.53
14	ATL25-ATL26	0.269	0.177	1.523	14.50
15	ATL43-ATL44	0.017	0.121	0.139	9.94
16	AUX5-AUX6	0.009	0.083	0.105	6.78
17	AAP13-AAP14	0.008	0.086	0.098	7.02
18	AAP28-AAP29	0.006	0.129	0.050	10.56
19	GAT14-GAT15	0.007	0.102	0.066	8.39
20	ProT2-ProT3	0.033	0.180	0.186	14.74
21	LHT5-LHT6	0.004	0.116	0.037	9.54
22	LHT7-LHT8	0.098	0.202	0.487	16.55
23	LHT14-LHT15	0.114	0.243	0.470	19.92

The history of selection acting on coding sequences can also be estimated according to Ka/Ks (Li et al., 1981). A pair of sequences will have Ka/Ks < 1 if one sequence has been under purifying selection but the other has been drifting neutrally, while Ka/Ks = 1 if both sequences are drifting neutrally and rarely, Ka/Ks > 1 at specific sites that are under positive selection (Juretic et al., 2005). Twenty-one pairs of all Ka/Ks ratios of duplicated *GmAAT* gene pairs were less than 0.6. A summary of Ka/Ks ratios was presented in Table 1. A total of 46 (24.3%) *AAT* genes in soybean were found located on duplicated chromosomal. The result suggested that all gene pairs have evolved mainly under the influence of purifying selection.

### Comparison of gene structures and conserved motifs among 189 AATs in soybean

**Figure 2:**
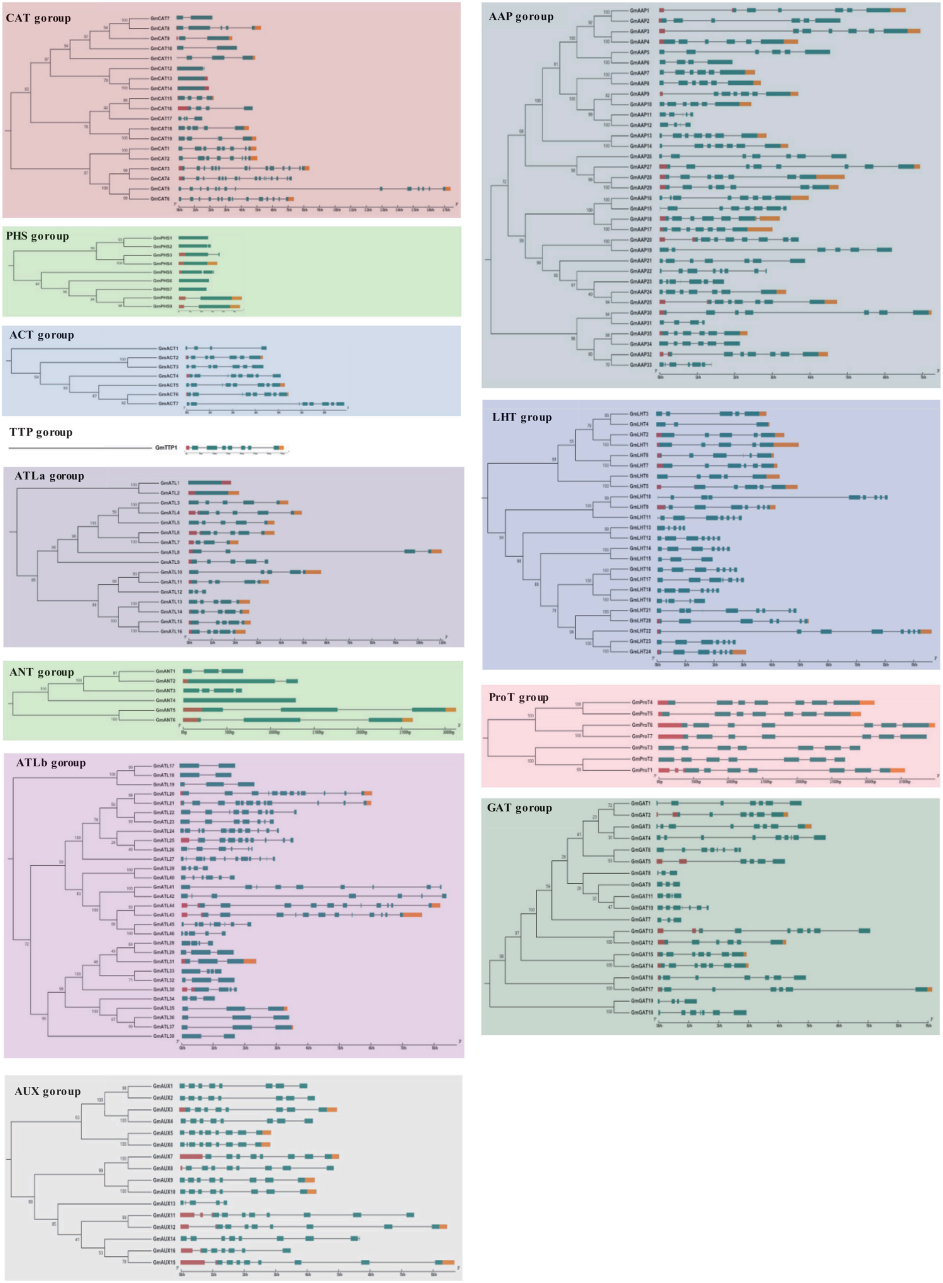
**Exon/intron organization of ***GmAAT*** genes in each subfamily**. Green boxes represent exons and block line represent introns. The untranslated regions (UTRs) are indicated by red and yellow boxes. The size of exons and introns can be estimated using the scale at the bottom.

It has been well studied that the intron/exon organizations, intron types and numbers are one of the representative imprints of the evolution within some gene families (Javelle et al., 2011; Du et al., 2012; Hudson and Hudson, 2015). We examined these features for the 189 *GmAAT* genes and observed that the same group shared similar exon/intron structures, e.g., intron and exon arrangement, such as *GmCAT13* and *GmCAT14, GmPHS8* and *GmPHS9, GmATL15* and *GmATL16, GmATL17* and *GmATL18* (Figure 2). In contrast, some members in the same cluster also showed variation in intron/exon organization. Obvious changes were found in the 5′-UTR or/and 3′-UTR of some genes as compared with their paralogs, such as *GmAAP24, GmATL13, GmATL36, GmAUX3, GmProT1*, et al. (Figure 2). The positions of exon and intron in the *AAT* genes of soybean are presented in Figure 2. Introns are absent from the open reading frames of nine *GmAAT* genes, and the number of introns in other coding sequences range from one to sixteen. Within subfamilies, most members share similar intron/exon structures and gene lengths. Gene structure analysis revealed that exon and intron positions and length were conserved within gene subfamilies, and this may indicate the close evolutionary relationships and the introduced classification of subfamilies. Together, these results indicate that intron/exon structure may have a role to result in functional conservation or diversification during long-term evolution among soybean *AAT* gene family.

The phylogenetic relationship and classification of GmAATs were further supported by motif analysis (Figure S1, Table 2). Twenty conserved motifs of GmAATs were captured by motif analysis using MEME software. We found that several motifs were widespread among GmAATs of the AAAP family, such as motif 4, 8, 11. In contrast, other motifs were specific to only one or two subfamilies. For example, motifs 1 and 2 were specific to the AUX subfamily, motifs 3 and 19 were specific to the AAP subfamily, motifs 14 and 18 were specific to the CAT subfamily, motifs 15 and 20 were specific to the ATLb subfamily, motif 16 occurred only in the ATLa subfamily, and motif 17 was found in only in subfamilies GAT and ProT. However, motif 8 was present in the ATLa, ATLb, AUX, AAP, GAT, ProT, LHT, and ANT subfamilies (Table 2).

**Table 2:** **The MEME motif sequences and lengths in GmAAT proteins**.

**Motif**	**Width**	**Conserved amino acid sequences**
1	152	KNHVIQWFEVLDGLLGPYWKALGLAFNCTFLLFGSVIQLIACASNIYYINDKLDKRTWTYIFGACCATTVFIPSFHNYRIWSFLGLGMTTYTAWYLAVAAILHGQVENVTHTGPTKLVLYFTGATNILYTFGGHAVTVEIMHAMWKPQKFKY
2	152	MLIHQFITFGFACTPLYFVWEKVIGMHDTKSICLRALARLPVVIPIWFLAIIFPFFGPINSAVGALLVSFTVYVIPACAHMLTYKSASARQNAAEKLPFFIPNWTAMYVVNAFVVVWVLVVGFGFGGWASMTNFIKQVDTFGLFAKCYQCPP
3	79	EAKTMKKATLISIAVTTTFYMLCGCMGYAAFGDAAPGNLLTGFGFYNPYWLIDIANAAIVIHLVGAYQVFSQPIFAFVE
4	39	KRTGTVWTASAHIITAVIGSGVLSLAWAIAQLGWIAGPI
5	41	RTVFVIITTVISMLLPFFNDILGVIGALGFWPLTVYFPVEM
6	43	FACIQIVLSQIPNFHKLWWLSIVAAVMSFAYSSIGLGLSVAKV
7	28	QAIGDIAFAYAYSNVLIEIQDTLKSSPP
8	28	VAYAVVALCYFPVGILGYWAFGNSVEDN
9	27	VEMHEMVPGKRFDRYHELGQHAFGEKL
10	28	LCGLVQYINLFGVAIGYTIAASTSMMAI
11	39	QKRTPRWSSRWIGMQILSVVCLIVSVAAAVGSVASIVLD
12	39	FFAYVGFDAVSTMAEETKNPARDIPIGLVGSMVITTLAY
13	39	EKPKWLIAMANMFVVIHVIGSYQIYAMPVFDMIETVMVK
14	164	CLLAVTLCLMQNYTDIDKDAPYSVAFSAVGMDWAKYIVAFGALKGMTTVLLVSAVGQARYLTHIARTHMMPPWFAHVDERTGTPMNATISMLAATAVIAFFTDLGILSNLLSISTLFIFMLVALALLVRRYYSSGLTTKENQVKLIVCLMLILGSSCAISAYWA
15	39	FAVICCYTATLMRYCFESREGITSYPDIGEAAFGKYGRI
16	71	ILQQWFGIHWWNSREFALLFTLVFVMLPLVLYKRVESLKYSSAVSTLLAVAFVGICCGLAITALVQGKTQT
17	39	YSLIGDTTNRLFGIFNAIPIIANTYGCGIVPEIQATLAP
18	55	PKAKEPKFWGVPLVPWIPSISIFINIFLLGSIDKDSFIRFGFWTVFLLVYYVFFG
19	20	NCYHKKGHEAPCKYGGNLYM
20	28	TLNMPKELVATKIAVWTTVVNPFTKYAL

### Phylogenetic analysis and multiple sequence alignment

**Figure 3:**
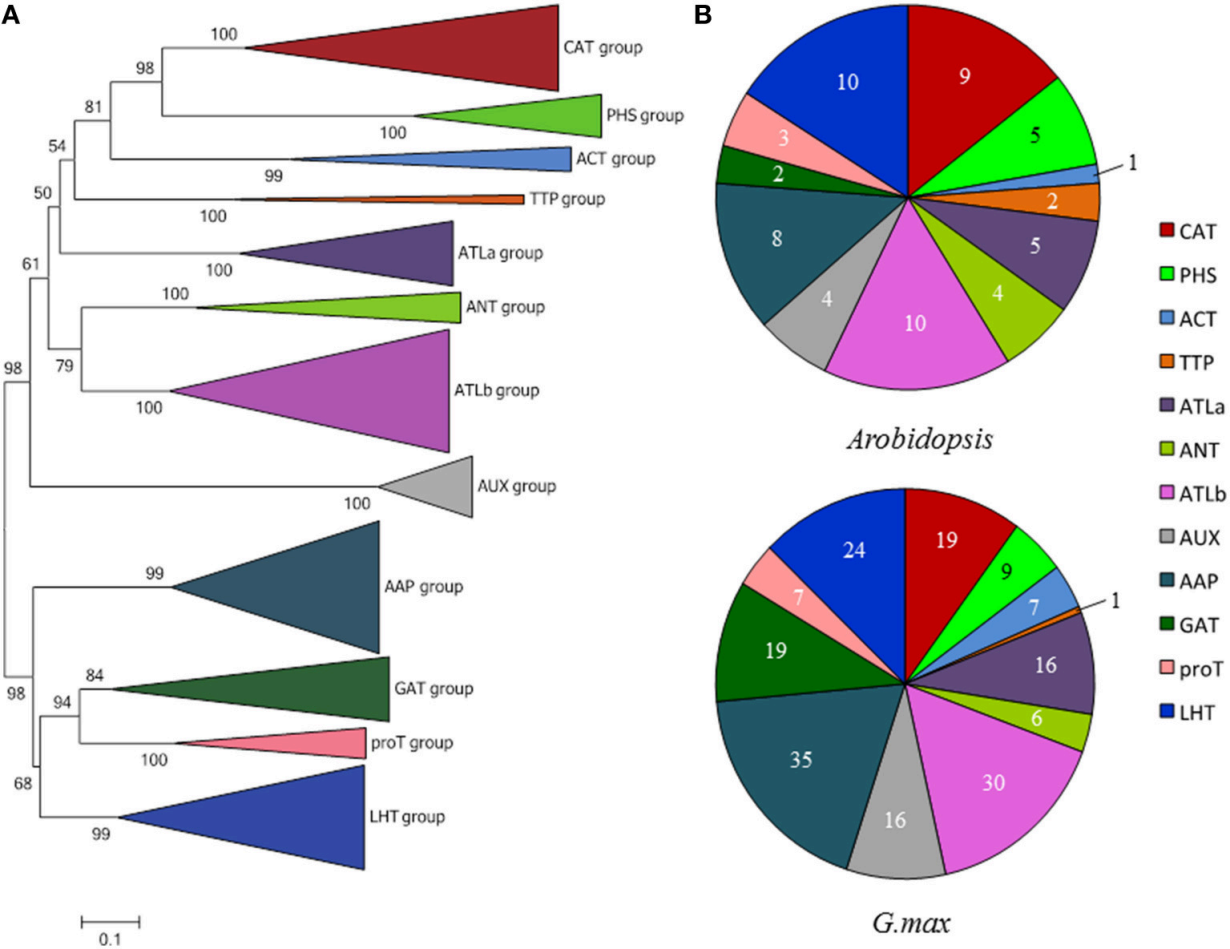
**Phylogenetic tree of the nucleotide sequences of ***AAT*** genes in soybean and ***Arabidopsis***. (A)** The tree was conducted based on the *AAT* gene nucleotide sequences using RAxML by 100 bootstrap replicates. The tree shows twelve major phylogenetic subfamilies indicated with different colors. **(B)** The different numbers were shown between *Arabidopsis* and soybean in each subfamily.

Our ML phylogenetic tree reveals 12 highly supported, distinct clades based on the similarities of their nucleotide sequence (Figure 3A, Figures S1, S2). This is a far greater number of *AAT* genes that are found in rice and *Arabidopsis*, which have 85 and 63 AATs, respectively. This could be caused by the experience of the recent twice whole genome duplication in soybean. We identified an AAAP family, which consists of 153 *GmAATs*, including eight distinct subfamilies comprising amino acid permeases (AAPs), lysine, histidine transporters (LHTs), proline transporters (ProTs), GABA transporters (GATs), auxin transporters (AUXs), aromatic and neutral amino acid transporters (ANTs) and amino acid transporter-like (ATL) subfamilies (Table S1). An ATL family consists of two clades or subfamilies: ATLa and ATLb. Our tree also supports an APC family that comprises 35 GmAATs and is subdivided into three distinct subfamilies: the cationic amino acid transporters (CATs), the amino acid/choline transporters (ACTs) and the polyamine H+-symporters (PHSs) (Table S1). Within soybean, we found large differences in the numbers of genes within the subfamilies (Figures S1, S2). In particular, we detected 35 genes in the GmAAP subfamily and only one in the GmTTP subfamily (Figure S1). Specifically, there were 19 GATs in soybean and 2 in *Arabidopsis*, 7 ACTs in soybean and 1 in *Arabidopsis*, 35 AAPs in soybean and 8 in *Arabidopsis*, 16 AUXs in soybean and 4 in *Arabidopsis*, 16 ATLas in soybean and 5 in *Arabidopsis*, and 30 ATLbs in soybean and 10 in *Arabidopsis* (Figure 3B). Nevertheless, our combined phylogenetic analysis of *AAT* genes in soybean and *Arabidopsis* revealed the monophyly of the subfamilies, which were shared between the two species (Figure 2, Figure S2).

**Figure 4:**
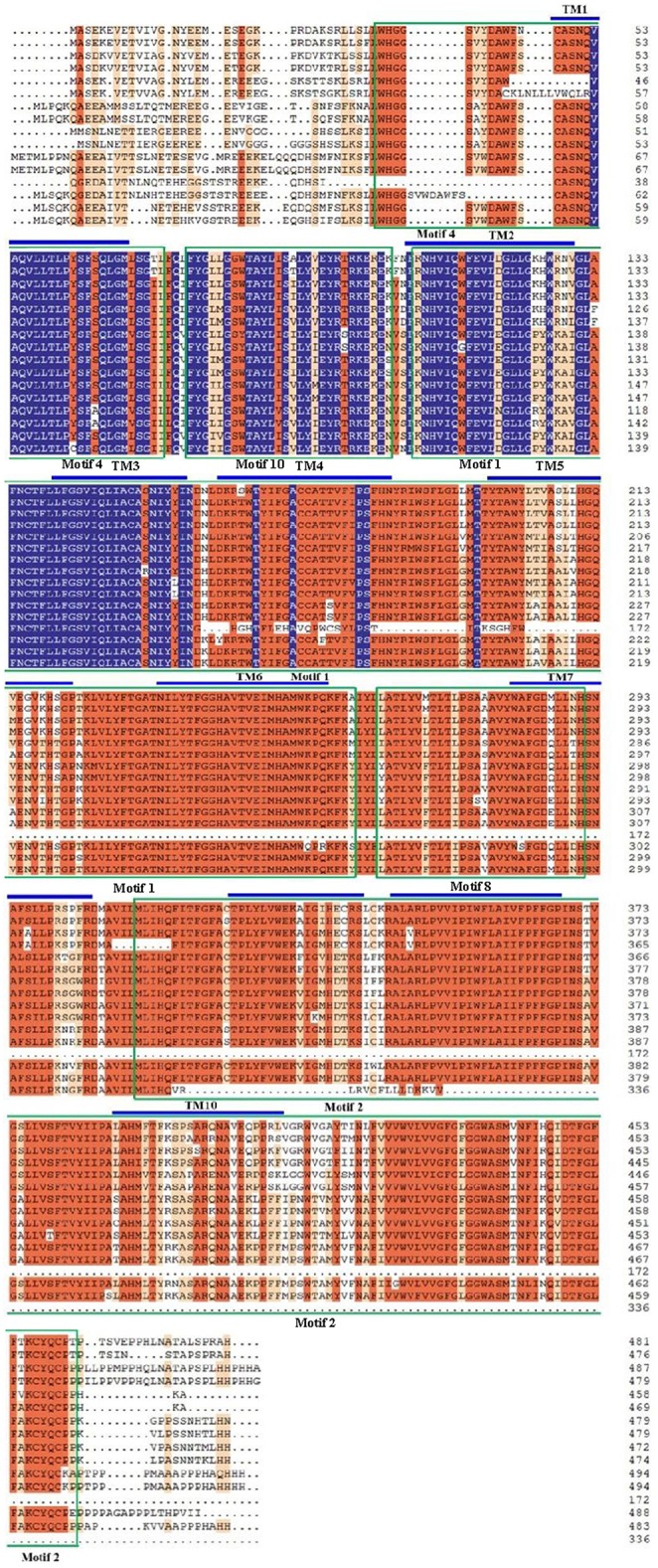
**Multiple sequence alignment and transmembrane region of AUX subfamily in soybean**. Identical (100%), conservative (75–99%), and block (50–74%) of similar amino acid residues are shaded in deep blue, brick red, and pale yellow, respectively. The transmembrane regions are marked by blue lines. The conserved motifs 1, 2, 4, 5, and 8 are orderly marked by green rectangles.

The alignments of the amino acid sequences of the GmAATs illustrated that TM regions were conserved within subfamilies. In addition, several TM regions of different members varied insignificantly both in length and amino acid composition. The alignment of the GmAUX members is shown in Figure 4 as an example. The protein sequences of the *GmAUX* genes are 75.04% similar. There are 5 conserved motifs in GmAUXs, including motifs 1, 2, 4, 8, and 10. TM1 was located in motif 4 (Figure 4). TM2, TM3, TM4, TM5, and TM6 were located in motif 1 (Figure 4). TM 8, TM9 and TM10 were located in motif 2 (Figure 4).

### The relationship between phylogenetic analysis and the expression patterns of *GmAAT* genes

**Figure 5:**
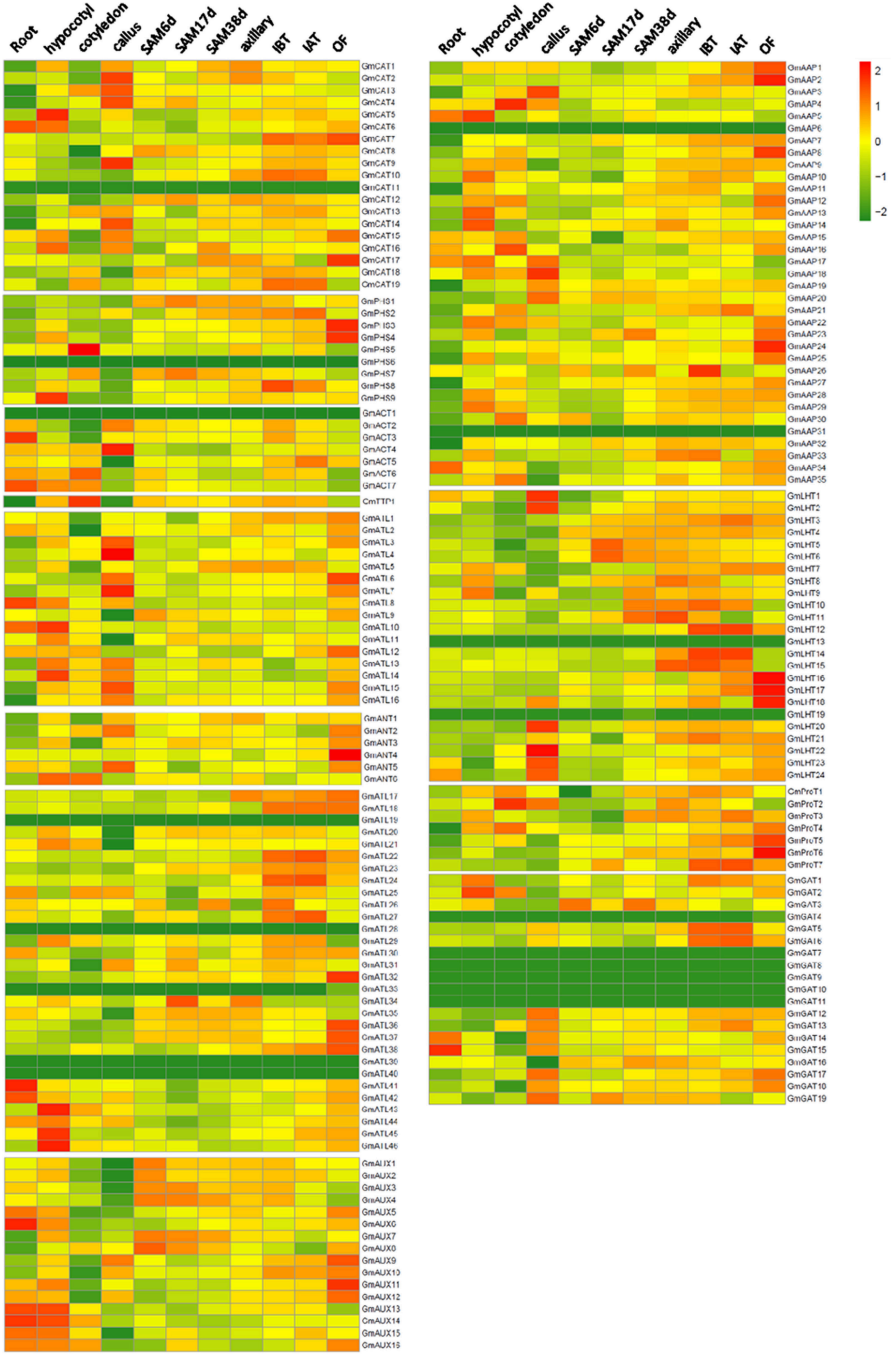
**Heat map representation of ***AAT*** genes in various tissues of soybean**. The RNA-seq relative expression data of 11 tissues was used to re-construct expression patterns of soybean *AAT* genes. Sources of samples are as follows: root, hypocotyl, cotyledon, callus, shoot apical meristem at 6, 17, and 38 day stage (referred to as SAM6d, SAM17d, SAM38d for convenience), as well as the axillary meristem (referred to as AM), inflorescences before and after the meiotic stage (referred to as IBM and IAM), and open flower (referred to as OF).

To determine expression patterns of *GmAAT* genes, we used publicly-available genome-wide transcript profiling data of soybean tissues as a resource (Wang et al., 2014). Results showed that soybean *AAT* genes were expressed in distinct patterns (Figure 5). In particular, 35 *AAT* genes in soybean showed less than twofold expression variation in different tissues, suggesting that they are not developmentally regulated at the transcription levels (Table S2). Multiple *GmAAT* genes were highly expressed in OF except the ACT group, which showed no expression in OF. *GmAAT* genes were constitutively expressed among several tissues, but others showed preferential expression in specific tissues. For example, *GmPHS3/GmANT4/GmATL37/GmATL38/GmAAP8/GmLHT17* were predominantly expressed in OF; *GmATL21* in hypocotyl; *GmATL41* in root; *GmLHT1/GmLHT2/GmLHT20/GmLHT22* in callus. Moreover, *GmATL6, GmAAP27, GmProT5*, and *GmLHT16* exhibited a highly tissue-specific expression pattern in OF while *GmATL7* and *GmLHT22* was highly concentrated in callus. Moreover, ten *GmAAT* genes had high expression levels in two different tissues.

The genes from GmACT subfamily were highly expressed in the root; especially *GmACT1* to *GmACT7*. The broad expression of these genes in roots may be related to regulation of developmental adaption to environmental stress conditions such as drought. Roots under water stress send chemical signals to shoots and also attempt to continue growth and obtain additional water resources through water foraging (Tardieu and Simonneau, 1998; Grant et al., 2010). Nine *GmAAT* genes showed no expression in one, three, five or six tissues. *GmANT4* was undetectable in hypocotyl, cotyledon, callus, SAM17d and axillary but highly expressed in OF; *GmLHT16* was not expressed in root, hypocotyl, cotyledon, callus, SAM17d, axillary but highly expressed in OF; *GmAUX13* was not expressed in callus, SAM17d and OF, but highly expressed in root and hypocotyl, respectively; *GmATL21* and *GmAUX2* were not expressed in callus but highly expressed in hypocotyl. Eighteen *GmAAT* genes were not detected in any of the tissues that we examined. These genes may be expressed in distinct tissue types or they may represent untranscribed pseudogenes.

Previous studies suggested that phylogenetic analysis provided clues on functional prediction of various genes. In general, members from the same subfamily shared similar exon/intron structures in exon length and the number of intron such as the GmAUX sbufamily; however, some members of the GmCAT, GmATLa, GmATLb, and GmAAP subfamilies (Figure 2, Table S1) had structural differences from other members. *AtLAX3* and *AtAUX1* were dominantly expressed in root and accelerated root formation laterally (Yang et al., 2006; Ugartechea-Chirino et al., 2010). It was also observed in our investigation that *GmAUX5, 6, 13, 14, 15*, and *16* were almost preferentially expressed in root. Similar expression patterns imply that these specificially expressed genes might play significant roles in root formation or development laterally. AAP members in *Arabidopsis* play critical roles in nutrient transport during seed development or long-distance transport of amino acids (Zhang et al., 2015). It was reported that *AtAAP1* localized to the plasma membrane of root epidermal cells and root hairs and involved in the uptake of amino acids into root cells (Lee et al., 2007). It was known that *AtAAP3* might be responsible for amino acid uptake from xylem (Okumoto et al., 2004). *AtAAP8* had been demonstrated that may be involved in amino acid uptake into the endosperm and supplying the developing embryo with amino acids during early embryogenesis (Schmidt et al., 2007). According to the previously reported, some of the AAPs were correlated with grain protein content (GPC), which is an important determinant of nutritional quality in cereals. *OsAAP6* was confirmed that could greatly enhance the absorption of several amino acids in root and has effects on the GPC (Peng et al., 2014). A *Vicia faba* amino acid permease, VfAAP1, which was reported improve plant nitrogen status and lead to higher seed protein contains by increasing seed sink strength for nitrogen (Rolletschek et al., 2005). Interestingly, several *GmAAP* genes were expressed in several specific tissues of soybean (Figure 5). The close relationship between sequence conservation and expression patterns may help us to select candidate genes that respond to diverse environmental stimuli or significant genes in developmental stages (Wang et al., 2012).

### Gene duplication and expression patterns of duplicated *AAT* genes

Events of gene duplication may serve as a critical mechanism for increasing the diversity of a gene family, especially through non-functionalization, sub-functionalization and neo-functionalization of the duplicated genes. Sub- and neo-functioning duplicates lead to functional diversity within families and may be expressed in a different tissue or at a different developmental stages than their progenitor (Bhattacharjee et al., 2015). Gene duplication and diversification events are well documented in *Arabidopsis* (Duarte et al., 2006). We observed that majority of duplicated *AAT* genes in soybean were differentially expressed within the tissue/organ/developmental stages. Based on gene expression patterns, we observed three types of functional variations in homologous gene pairs in soybean. For instance, we observed sub-functionalization or neo-functionalization in the *GmATL25/GmATL26* gene pair. Specifically, *GmATL25* but not *GmATL26* was expressed in the cotyledons and the callus, while *GmATL26* but not *GmATL25* was expressed in the SAM6D and SAM38D. In the *GmGAT16/ GmGAT17* gene pair, *GmGAT16* expression decreased to basal level in the callus, IAT and OF compared to *GmGAT17*. In the *GmCAT7/GmCAT8* gene pair, *GmCAT7* was only expressed in IBT, IAT and OF compared to *GmCAT8*. In the *GmANT5*/*GmANT6* gene pair, *GmANT5* was expressed only in the callus and OF, but *GmANT6* was expressed only in hypocotyls and cotyledons. In the *GmProT2/GmProT3* gene pair, we detected high expression levels of *GmProT2* in cotyledons and the callus, but *GmProT3* was not expressed in these two tissues. The *GmAAP2*/*GmAAP3* gene pair, exhibited identical expression patters in all ten tissues except in the callus and OF. The phenomenon of non-functionalization was exhibited in the *GmGAT2/GmGAT4/GmGAT5* duplication event. In particular, *GmGAT2* and *GmGAT5* were expressed in the hypocotyls, cotyledons, IBT, IAT, and OF, but *GmGAT4* did not show expression in most of the tissues that we surveyed (Figure 5). These observations imply that the evolutionary fate of *AAT* genes in soybean have been strongly affected by gene duplication events. Overall, our results show that purifying selection has contributed to retention and maintenance of duplicated gene pairs during evolution. Moreover, expression profiling of duplicated soybean AAT proteins highlighted that majority of them have undergone sub-functionalization. Such observations are consistent with other plant species, where closely related genes have diverse expression patterns.

### Expression profiles of *AAT* genes and functional diversity of duplicated pairs in soybean

**Figure 6:**
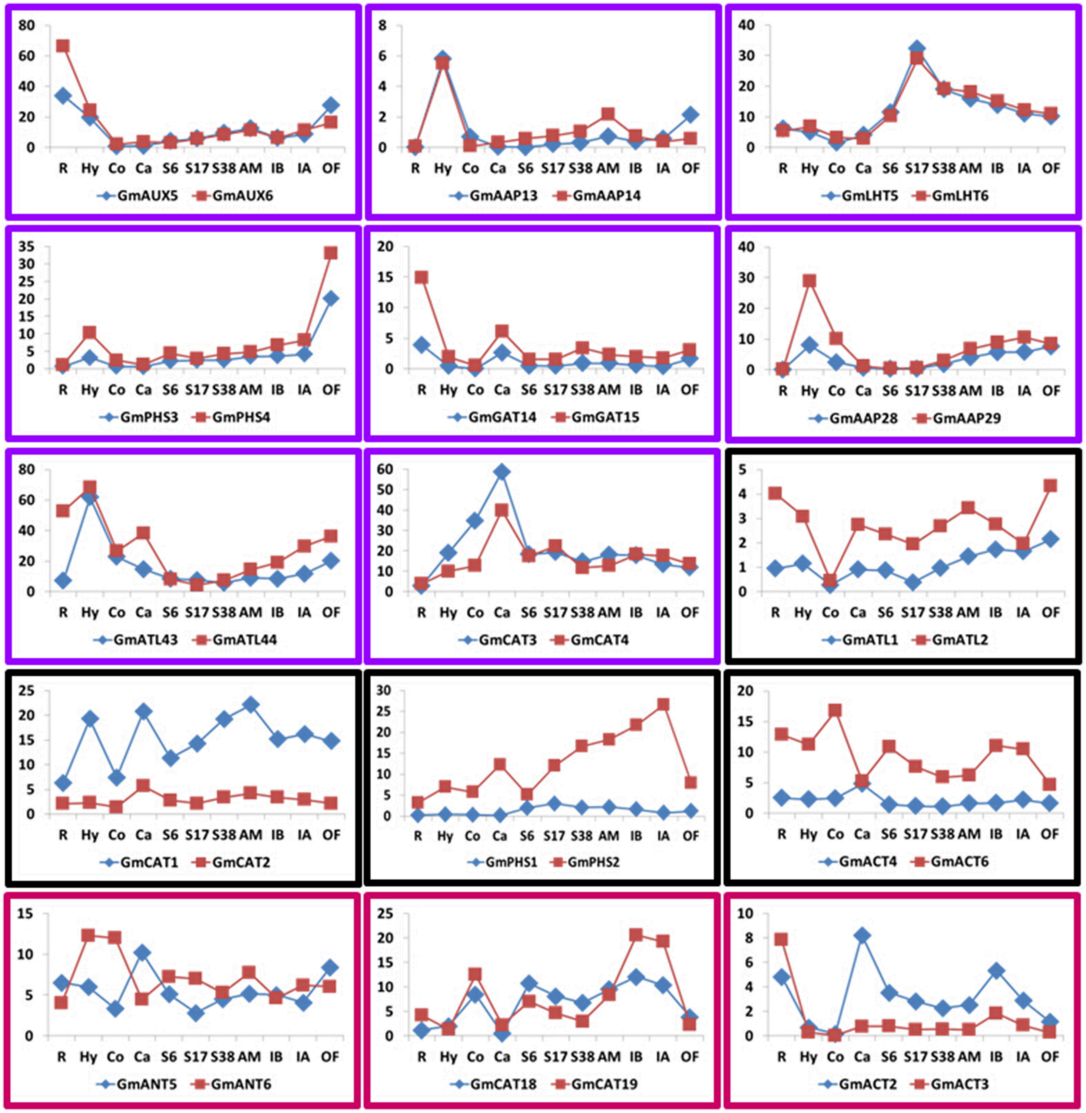
**Three trends of expression patterns of duplicated ***AAT*** gene pairs in soybean**. X-axis indicates representative tissues, organs and Y-axis represents scale. Purple box: two copies have the same expression pattern in almost 11 tissues. Black box: one duplicate was expressed at higher levels than the other in all tissues. Fuchsia box: the two copies have the diversely expression patterns in different tissues. R, root; Hy, hypocotyl; Co, cotyledon; Ca, callus; S6, SAM6d; S17, SAM17d; S38, SAM38d; AM, axillary meristem; IB, inflorescences before the meiotic stage; IA, inflorescences after the meiotic stage; OF, open flower.

We investigated the expression profiles of *AAT* genes using public soybean expression data and found that most *AAT* genes are widely expressed, suggesting that these soybean *AAT* genes remained after WGD events are likely functional (Figure 6). According to the expression patterns of soybean *AAT* genes as related to their *Arabidopsis* orthologs, we hypothesize that these genes may also have potential functions in soybean. We also found that soybean and *Arabidopsis* have 23 and 15 duplicated *AAT* gene pairs, respectively. Among the 23 duplicated gene pairs, 15 of them are expressed in two copies and were defined to three types regarding on expression patterns (Figure 6).

The first type is that two copies have the same expression pattern in almost eleven tissues (Figure 6, purple boxes). For example, The *Arabidopsis AtAAP6* gene corresponds to the soybean *AAP28* and *AAP29*. *AtAAP6* might be serve a different role either in cooperating with the lower affinity systems to acquire amino acids in the low concentration range, as a system responsible for aspartate transport or as a uptake system from the xylem (Fischer et al., 2002). *GmLHT5/GmLHT6, GmAAP13/GmAAP14* were the highest expression in SAM17d or hypocotyl relative to the other tissues, suggesting that these genes may undertake significant functions in special developmental stage of soybean. The second type is that one duplicate was expressed at higher levels than the other in all tissues (Figure 6, black boxes), implying that the former has a stronger function than the latter. One copy had wide-range high level expression indicating that it my play important roles in regulating broad developmental or reproductive stages. The third type is that the two copies have the diversely expression patterns in different tissues (Figure 6, fuchsia boxes). For example, *GmANT5* and *GmANT6* corresponded to *Arabidopsis ANT1*, which was induced by high concentrations of nitrate and expressed in all organs with highest abundance in cauline leaves and flowers (Chen et al., 2001; Liu and Bush, 2006). The *GmANT5* and *GmANT6* pairs in soybean are both expressed at the same stage.

The different expression patterns of 15 duplicated gene pairs in different tissues imply possible function redundancy or functional diversification, respectively (Innan and Kondrashov, 2010). Diverse expression profiles in different tissues of the duplicates may finally result in subfunctionalization or neofunctionalization, suggesting different functions or silencing of one copy (Liu et al., 2011), or similar expression levels in developmental stages or tissues implying functional conservation (Li et al., 2012; Zhang and Ma, 2012).

## Conclusion

We identified 189 *AAT* genes in soybean by means of a genome-wide analysis. The soybean genome had more *AAT* genes than the genomes of *Arabidopsis* (63) or rice (85). The great expansion of the *AAT* genes in soybean is probably the result of WGD and tandomly duplications that occurred during evolutionary history of the family Fabaceae. We have presented an overview of the chromosomal distributions, gene structures, duplication patterns, phylogeny, and conserved motifs in soybean AATs. Additionally, we have annotated *AAT* genes in soybean based on data from the rice and *Arabidopsis* genomes and we have comprehensively assessed expression patterns of AATs in specific soybean tissues. Our study provides a comprehensive framework for future studies of *AAT* genes in soybeans.

## Author contributions

LC, YW, and LW designed the whole experiments; LC and HY wrote the manuscript; RR analyzed the soybean genome data and constructed the AAT phylogenetic trees; SZ and YH annotated the AAT genes on chromosomes and calculated the duplication date; QZ and DK analyzed the expression data.

### Conflict of interest statement

The authors declare that the research was conducted in the absence of any commercial or financial relationships that could be construed as a potential conflict of interest.

## Supplementary material

Table S1 **The general information and sequence characterization of 189 *GmAAT* genes**.

Table S2 **The expression data of *GmAAT* genes in various organs**.

Figure S1 **Phylogenetic tree of the nucleotide sequences of *AAT* genes in soybean**.

Figure S2 **Phylogenetic tree of the nucleotide sequences of *AAT* genes in soybean and *Arabidopsis***.
