# Supplementary material for: Genome-Wide Identification, Classification, and Expression Analysis of Amino Acid Transporter Gene Family in Glycine Max
Source: Front Plant Sci. 2016 Apr 20;7:515. doi: 10.3389/fpls.2016.00515 (PMC4837150; doi:10.3389/fpls.2016.00515)
Supplement: Table S1 — The general information and sequence characterization of 189 GmAAT genes. [file Table1.DOC]

**Table S1. The general information and sequence characterization of 189 *GmAAT* genes.**

| **S.N.** | | **Gene^a^** | **Locus^b^** | | | **Gene Structure** | | | **ORF(bp)^e^** | **Protein^f^** | | | **TM region^g^** |
| --- | --- | --- | --- | --- | --- | --- | --- | --- | --- | --- | --- | --- | --- |
|  |  |  |  |  |  | **Length(bp)^c^** | | **Intron^d^** |  | **Size (aa)** | **MW(d)** | **pI** |  |
|  | | CAT group |  | | |  | |  |  |  |  |  |  |
| 1 | | *GmCAT1* | Glyma09g37700 | | | 4805 | | 7 | 1677 | 558 | 59520.89 | 7.55 | 13 |
| 2 | | *GmCAT 2* | Glyma18g48860 | | | 4855 | | 7 | 1560 | 519 | 55427.9 | 7.54 | 11 |
| 3 | | *GmCAT 3* | Glyma16g04050 | | | 8072 | | 13 | 1923 | 640 | 68346.96 | 6.09 | 14 |
| 4 | | *GmCAT 4* | Glyma19g29440 | | | 7302 | | 16 | 1902 | 633 | 67674.64 | 7.14 | 14 |
| 5 | | *GmCAT 5* | Glyma05g11410 | | | 17589 | | 13 | 1923 | 640 | 67557.93 | 5.50 | 14 |
| 6 | | *GmCAT 6* | Glyma19g29450 | | | 7433 | | 13 | 1905 | 634 | 67620.14 | 5.88 | 15 |
| 7 | | *GmCAT 7* | Glyma05g30510 | | | 2306 | | 1 | 1803 | 600 | 65592.45 | 6.38 | 14 |
| 8 | | *GmCAT 8* | Glyma08g13660 | | | 5469 | | 4 | 2229 | 742 | 81112.59 | 8.74 | 14 |
| 9 | | *GmCAT 9* | Glyma08g13640 | | | 3589 | | 2 | 1809 | 602 | 65946.83 | 7.10 | 13 |
| 10 | | *GmCAT 10* | Glyma09g10300 | | | 3879 | | 1 | 1701 | 567 | 62482.34 | 8.44 | 14 |
| 11 | | *GmCAT 11* | Glyma09g21070 | | | 4853 | | 3 | 1734 | 577 | 63021.09 | 8.71 | 14 |
| 12 | | *GmCAT 12* | Glyma09g01230 | | | 1774 | | 1 | 1710 | 569 | 62303.99 | 8.77 | 12 |
| 13 | | *GmCAT 13* | Glyma09g05580 | | | 1937 | | 0 | 1758 | 585 | 64761.73 | 8.98 | 14 |
| 14 | | *GmCAT 14* | Glyma09g05540 | | | 2033 | | 0 | 1770 | 589 | 65333.34 | 8.96 | 14 |
| 15 | | *GmCAT 15* | Glyma17g15840 | | | 5182 | | 3 | 1296 | 431 | 47097.0 | 9.35 | 8 |
| 16 | | *GmCAT 16* | Glyma05g05510 | | | 4833 | | 3 | 1299 | 432 | 47710.7 | 7.18 | 10 |
| 17 | | *GmCAT 17* | Glyma11g04100 | | | 1527 | | 3 | 861 | 287 | 32382.5 | 7.29 | 6 |
| 18 | | *GmCAT 18* | Glyma20g24620 | | | 4365 | | 4 | 1764 | 587 | 63148.47 | 7.59 | 15 |
| 19 | | *GmCAT 19* | Glyma10g42440 | | | 4823 | | 4 | 1506 | 501 | 54009.2 | 9.21 | 13 |
|  | | PHS group |  | | |  | |  |  |  |  |  |  |
| 20 | | *GmPHS1* | Glyma14g35520 | | | 1362 | | 0 | 1362 | 454 | 50175.6 | 9.79 | 10 |
| 21 | | *GmPHS 2* | Glyma02g37200 | | | 1491 | | 1 | 1449 | 482 | 53013.3 | 8.69 | 8 |
| 22 | | *GmPHS 3* | Glyma05g28810 | | | 1886 | | 1 | 1113 | 370 | 41200.4 | 9.00 | 8 |
| 23 | | *GmPHS 4* | Glyma08g11960 | | | 1788 | | 0 | 1089 | 362 | 40284.7 | 8.53 | 8 |
| 24 | | *GmPHS 5* | Glyma01g45410 | | | 1631 | | 2 | 1404 | 467 | 51951.8 | 5.18 | 10 |
| 25 | | *GmPHS 6* | Glyma20g24020 | | | 1389 | | 0 | 1389 | 462 | 51361.1 | 9.25 | 9 |
| 26 | | *GmPHS 7* | Glyma16g17460 | | | 1287 | | 0 | 1287 | 429 | 47597.0 | 6.52 | 10 |
| 27 | | *GmPHS 8* | Glyma01g35320 | | | 2931 | | 1 | 1461 | 486 | 53772.9 | 8.52 | 12 |
| 28 | | *GmPHS 9* | Glyma09g34750 | | | 2844 | | 1 | 1461 | 486 | 53876.9 | 7.49 | 12 |
|  | | ACT group |  | | |  | |  |  |  |  |  |  |
| 29 | | *GmACT1* | Glyma10g41570 | | | 3558 | | 3 | 534 | 177 | 19475.0 | 5.63 | 0 |
| 30 | | *GmACT2* | Glyma04g38710 | | | 3396 | | 6 | 1539 | 512 | 56291.9 | 8.62 | 10 |
| 31 | | *GmACT3* | Glyma06g16280 | | | 3395 | | 6 | 1584 | 527 | 57897.3 | 8.23 | 10 |
| 32 | | *GmACT4* | Glyma20g25660 | | | 4173 | | 7 | 1593 | 530 | 57420.6 | 8.76 | 12 |
| 33 | | *GmACT5* | Glyma20g25680 | | | 4357 | | 7 | 1560 | 519 | 56588.4 | 8.17 | 12 |
| 34 | | *GmACT6* | Glyma10g41560 | | | 4497 | | 7 | 1563 | 520 | 56357.5 | 9.18 | 10 |
| 35 | | *GmACT7* | Glyma20g25670 | | | 6963 | | 7 | 1548 | 515 | 55792.7 | 8.40 | 10 |
|  | | TTP group |  | | |  | |  |  |  |  |  |  |
| 36 | | *GmTTP1* | Glyma16g27100 | | | 3733 | | 7 | 1383 | 460 | 50138.8 | 8.93 | 11 |
|  | | ATLa group |  | | |  | |  |  |  |  |  |  |
| 37 | | *GmATL1* | Glyma15g00870 | | | 1863 | | 0 | 1458 | 485 | 52946.4 | 6.00 | 11 |
| 38 | | *GmATL2* | Glyma13g44450 | | | 2207 | | 0 | 1458 | 485 | 52943.3 | 5.20 | 11 |
| 39 | | *GmATL3* | Glyma19g05000 | | | 4374 | | 4 | 1395 | 464 | 50151.5 | 8.22 | 11 |
| 40 | | *GmATL4* | Glyma13g06930 | | | 5028 | | 5 | 1398 | 465 | 50327.8 | 7.85 | 11 |
| 41 | | *GmATL5* | Glyma19g04990 | | | 3765 | | 4 | 1392 | 463 | 49953.2 | 7.21 | 10 |
| 42 | | *GmATL6* | Glyma08g28190 | | | 3753 | | 5 | 1401 | 466 | 50364.0 | 8.29 | 11 |
| 43 | | *GmATL7* | Glyma18g51220 | | | 2136 | | 3 | 813 | 270 | 29426.4 | 7.38 | 7 |
| 44 | | *GmATL8* | Glyma06g29640 | | | 11127 | | 3 | 1203 | 400 | 43604.6 | 7.10 | 10 |
| 45 | | *GmATL9* | Glyma14g10260 | | | 3498 | | 4 | 1215 | 404 | 44468.5 | 7.10 | 10 |
| 46 | | *GmATL10* | Glyma14g05910 | | | 5805 | | 4 | 1332 | 443 | 47828.7 | 6.87 | 10 |
| 47 | *GmATL11* | | Glyma02g42810 | | | 3517 | | 4 | 1209 | 402 | 43452.8 | 8.86 | 10 |
| 48 | *GmATL12* | | Glyma14g05900 | | | 748 | | 2 | 486 | 161 | 17781.7 | 6.67 | 3 |
| 49 | *GmATL13* | | Glyma02g42800 | | | 2675 | | 4 | 1305 | 434 | 47017.3 | 8.13 | 10 |
| 50 | *GmATL14* | | Glyma14g05890 | | | 2666 | | 4 | 1299 | 432 | 46999.3 | 8.31 | 10 |
| 51 | | *GmATL15* | Glyma18g02580 | | | 2725 | | 4 | 1311 | 436 | 46923.1 | 8.89 | 10 |
| 52 | | *GmATL16* | Glyma11g35830 | | | 2491 | | 4 | 1311 | 436 | 46966.2 | 8.69 | 10 |
|  | ATLb group | | | |  |  | |  |  |  |  |  |  |
| 53 | *GmATL17* | | | | Glyma11g36880 | | 1737 | 1 | 1120 | 374 | 40528.9 | 9.59 | 10 |
| 54 | *GmATL18* | | | | Glyma18g00780 | | 1620 | 1 | 1106 | 369 | 39768.1 | 9.14 | 10 |
| 55 | *GmATL19* | | | | Glyma05g28160 | | 2440 | 2 | 1260 | 419 | 45684.9 | 9.07 | 10 |
| 56 | *GmATL20* | | | | Glyma02g30960 | 6286 | | 11 | 1665 | 554 | 61085.8 | 4.78 | 11 |
| 57 | *GmATL21* | | | | Glyma10g12290 | 6258 | | 11 | 1581 | 526 | 57503.0 | 8.19 | 9 |
| 58 | *GmATL22* | | | | Glyma19g31090 | 3793 | | 8 | 1339 | 447 | 49257.2 | 5.15 | 8 |
| 59 | *GmATL23* | | | | Glyma03g28370 | 2941 | | 6 | 1152 | 383 | 42772.2 | 5.94 | 6 |
| 60 | *GmATL24* | | | | Glyma14g15070 | 3225 | | 11 | 1314 | 437 | 49456.1 | 6.36 | 7 |
| 61 | *GmATL25* | | | | Glyma01g42750 | 3554 | | 9 | 1146 | 381 | 41690.9 | 8.32 | 8 |
| 62 | *GmATL26* | | | | Glyma11g02700 | 2335 | | 6 | 732 | 244 | 27754.6 | 7.62 | 3 |
| 63 | *GmATL27* | | | | Glyma09g24210 | 3093 | | 11 | 1125 | 375 | 43082.7 | 5.87 | 5 |
| 64 | *GmATL28* | | | | Glyma01g27180 | 980 | | 3 | 654 | 218 | 24086.7 | 8.17 | 5 |
| 65 | *GmATL29* | | | | Glyma18g49420 | 1662 | | 2 | 1290 | 429 | 46777.4 | 6.71 | 10 |
| 66 | *GmATL30* | | | | Glyma09g39320 | 1797 | | 4 | 936 | 311 | 34648.9 | 9.72 | 7 |
| 67 | *GmATL31* | | | | Glyma09g37270 | 2351 | | 2 | 1281 | 426 | 45933.5 | 7.44 | 11 |
| 68 | *GmATL32* | | | | Glyma09g37260 | 1656 | | 2 | 1230 | 409 | 44148.7 | 8.05 | 11 |
| 69 | *GmATL33* | | | | Glyma18g11330 | 1301 | | 3 | 1012 | 338 | 37432.5 | 7.81 | 7 |
| 70 | *GmATL34* | | | | Glyma11g29030 | 1037 | | 2 | 742 | 248 | 26873.8 | 9.78 | 6 |
| 71 | *GmATL35* | | | | Glyma18g06650 | 3452 | | 2 | 1308 | 435 | 46860.5 | 7.47 | 11 |
| 72 | *GmATL36* | | | | Glyma11g29080 | 3518 | | 2 | 1314 | 437 | 47202.8 | 7.02 | 11 |
| 73 | *GmATL37* | | | | Glyma11g29050 | 3509 | | 2 | 1308 | 435 | 46888.6 | 7.75 | 11 |
| 74 | *GmATL38* | | | | Glyma20g04840 | 1671 | | 1 | 951 | 317 | 34943.7 | 8.74 | 8 |
| 75 | *GmATL39* | | | | Glyma1675s00200 | 874 | | 3 | 528 | 176 | 19508.6 | 7.49 | 5 |
| 76 | *GmATL40* | | | | Glyma18g40080 | 1751 | | 5 | 726 | 241 | 27253.9 | 8.19 | 6 |
| 77 | *GmATL41* | | | | Glyma02g19430 | 8564 | | 8 | 1290 | 430 | 46726.3 | 7.39 | 7 |
| 78 | *GmATL42* | | | | Glyma10g15130 | 8724 | | 6 | 958 | 320 | 34690.1 | 6.68 | 6 |
| 79 | *GmATL43* | | | | Glyma20g32260 | 7571 | | 12 | 1635 | 544 | 58633.1 | 6.27 | 10 |
| 80 | *GmATL44* | | | | Glyma10g35280 | 8510 | | 11 | 1614 | 537 | 58034.5 | 6.24 | 9 |
| 81 | *GmATL45* | | | | Glyma09g26880 | 2197 | | 7 | 762 | 253 | 28875.3 | 9.85 | 3 |
| 82 | *GmATL46* | | | | Glyma16g08770 | 1402 | | 4 | 564 | 187 | 21211.3 | 8.06 | 3 |
|  | | ANT group | | |  |  | |  |  |  |  |  |  |
| 83 | | *GmANT1* | | | Glyma09g33430 | 688 | | 2 | 511 | 171 | 18782.7 | 7.29 | 2 |
| 84 | | *GmANT2* | | | Glyma11g09190 | 1951 | | 1 | 1125 | 374 | 40563.6 | 8.46 | 8 |
| 85 | | *GmANT3* | | | Glyma01g36250 | 728 | | 3 | 606 | 201 | 22254.4 | 7.81 | 4 |
| 86 | | *GmANT4* | | | Glyma09g33030 | 1287 | | 0 | 1287 | 428 | 46986.9 | 6.50 | 11 |
| 87 | | *GmANT5* | | | Glyma19g39060 | 3127 | | 2 | 1269 | 422 | 45513.1 | 7.11 | 10 |
| 88 | | *GmANT6* | | | Glyma03g36410 | 2630 | | 2 | 1062 | 353 | 38472.2 | 5.06 | 8 |
|  | | AUX group | |  | |  | |  |  |  |  |  |  |
| 89 | | *GmAUX1* | Glyma06g00690 | | | 4049 | | 7 | 1446 | 481 | 54322.0 | 8.86 | 10 |
| 90 | | *GmAUX2* | Glyma04g00640 | | | 4288 | | 7 | 1431 | 476 | 53787.3 | 9.18 | 10 |
| 91 | | *GmAUX3* | Glyma11g11310 | | | 5004 | | 7 | 1467 | 488 | 55130.1 | 9.21 | 10 |
| 92 | | *GmAUX4* | Glyma12g03490 | | | 4217 | | 7 | 1443 | 480 | 54119.9 | 9.21 | 10 |
| 93 | | *GmAUX5* | Glyma06g11540 | | | 2892 | | 7 | 1377 | 458 | 51825.6 | 8.88 | 11 |
| 94 | | *GmAUX6* | Glyma04g43150 | | | 2857 | | 8 | 1410 | 469 | 53037.9 | 9.04 | 10 |
| 95 | | *GmAUX7* | Glyma11g35080 | | | 5058 | | 8 | 1440 | 479 | 54180.6 | 8.34 | 10 |
| 96 | | *GmAUX8* | Glyma18g03280 | | | 4875 | | 8 | 1440 | 479 | 54199.7 | 8.64 | 10 |
| 97 | | *GmAUX9* | Glyma14g06600 | | | 4283 | | 7 | 1419 | 472 | 54143.7 | 8.50 | 10 |
| 98 | | *GmAUX10* | Glyma02g42290 | | | 4333 | | 7 | 1425 | 474 | 53442.9 | 8.57 | 10 |
| 99 | | *GmAUX11* | Glyma07g17810 | | | 7865 | | 9 | 1485 | 494 | 55871.3 | 8.04 | 10 |
| 100 | | *GmAUX12* | Glyma18g42640 | | | 8481 | | 8 | 1485 | 494 | 55895.4 | 8.04 | 10 |
| 101 | | *GmAUX13* | Glyma01g28060 | | | 1487 | | 4 | 516 | 172 | 19424.7 | 6.62 | 2 |
| 102 | | *GmAUX14* | Glyma03g09140 | | | 5706 | | 7 | 1467 | 488 | 55145.7 | 8.03 | 10 |
| 103 | | *GmAUX15* | Glyma03g09100 | | | 8709 | | 8 | 1452 | 483 | 54583.2 | 8.43 | 10 |
| 104 | | *GmAUX16* | Glyma01g28310 | | | 3504 | | 6 | 1011 | 336 | 38167.8 | 8.45 | 6 |
|  | | AAP group |  | | |  | |  |  |  |  |  |  |
| 105 | | *GmAAP1* | Glyma06g16340 | | | 6648 | | 6 | 1410 | 469 | 51389.4 | 8.57 | 10 |
| 106 | | *GmAAP2* | Glyma04g38650 | | | 6772 | | 5 | 1461 | 486 | 53285.1 | 8.73 | 9 |
| 107 | | *GmAAP3* | Glyma04g38640 | | | 7040 | | 6 | 1464 | 487 | 53806.1 | 6.76 | 9 |
| 108 | | *GmAAP4* | Glyma06g16350 | | | 7876 | | 5 | 1596 | 531 | 58461.8 | 7.10 | 10 |
| 109 | | *GmAAP5* | Glyma05g32810 | | | 4405 | | 5 | 1455 | 484 | 53140.4 | 8.92 | 9 |
| 110 | | *GmAAP6* | Glyma08g00460 | | | 1885 | | 3 | 1146 | 381 | 42314.4 | 9.51 | 7 |
| 111 | | *GmAAP7* | Glyma13g10070 | | | 2464 | | 5 | 1440 | 479 | 52719.9 | 8.69 | 9 |
| 112 | | *GmAAP8* | Glyma14g24370 | | | 2618 | | 5 | 1440 | 479 | 52563.8 | 8.55 | 9 |
| 113 | | *GmAAP9* | Glyma04g42520 | | | 3736 | | 6 | 1464 | 487 | 53603.7 | 8.23 | 10 |
| 114 | | *GmAAP10* | Glyma06g12270 | | | 2463 | | 5 | 1464 | 487 | 53551.7 | 8.23 | 9 |
| 115 | | *GmAAP11* | Glyma02g34510 | | | 898 | | 4 | 417 | 139 | 15679.4 | 7.09 | 1 |
| 116 | | *GmAAP12* | Glyma04g32730 | | | 791 | | 3 | 417 | 138 | 15729.7 | 9.85 | 2 |
| 117 | | *GmAAP13* | Glyma11g11440 | | | 2877 | | 6 | 1416 | 471 | 51981.5 | 9.37 | 9 |
| 118 | | *GmAAP14* | Glyma12g03580 | | | 3467 | | 6 | 1416 | 471 | 52094.7 | 9.45 | 9 |
| 119 | | *GmAAP15* | Glyma08g44940 | | | 3410 | | 7 | 1410 | 469 | 51303.2 | 6.41 | 9 |
| 120 | | *GmAAP16* | Glyma18g07970 | | | 3845 | | 7 | 1389 | 462 | 50667.5 | 5.13 | 9 |
| 121 | | *GmAAP17* | Glyma02g47370 | | | 3031 | | 6 | 1434 | 477 | 52160.0 | 7.32 | 9 |
| 122 | | *GmAAP18* | Glyma14g01370 | | | 2963 | | 6 | 1323 | 440 | 48644.1 | 8.90 | 9 |
| 123 | | *GmAAP19* | Glyma02g47350 | | | 6252 | | 5 | 1311 | 436 | 48178.0 | 8.50 | 11 |
| 124 | | *GmAAP20* | Glyma14g01410 | | | 3583 | | 8 | 1320 | 439 | 48746.4 | 8.60 | 10 |
| 125 | | *GmAAP21* | Glyma18g07980 | | | 3914 | | 6 | 1386 | 461 | 50810.3 | 8.90 | 9 |
| 126 | | *GmAAP22* | Glyma17g32240 | | | 2643 | | 7 | 714 | 237 | 25656.4 | 7.80 | 4 |
| 127 | | *GmAAP23* | Glyma19g07580 | | | 1592 | | 6 | 968 | 323 | 36236.3 | 8.84 | 8 |
| 128 | | *GmAAP24* | Glyma18g08000 | | | 3270 | | 6 | 1386 | 461 | 50819.3 | 8.90 | 9 |
| 129 | | *GmAAP25* | Glyma08g44930 | | | 4775 | | 7 | 1386 | 461 | 50991.5 | 8.80 | 10 |
| 130 | | *GmAAP26* | Glyma10g40130 | | | 5044 | | 6 | 1371 | 456 | 50062.2 | 9.27 | 9 |
| 131 | | *GmAAP27* | Glyma17g26590 | | | 7030 | | 7 | 1515 | 504 | 55867.1 | 9.23 | 10 |
| 132 | | *GmAAP28* | Glyma06g09470 | | | 4772 | | 6 | 1440 | 479 | 53313.4 | 8.88 | 10 |
| 133 | | *GmAAP29* | Glyma04g09310 | | | 4612 | | 6 | 1440 | 479 | 53276.3 | 8.80 | 10 |
| 134 | | *GmAAP30* | Glyma14g22120 | | | 7338 | | 6 | 1383 | 460 | 50258.2 | 8.92 | 11 |
| 135 | | *GmAAP31* | Glyma14g21870 | | | 1162 | | 3 | 513 | 170 | 18828.9 | 9.47 | 3 |
| 136 | | *GmAAP32* | Glyma06g09270 | | | 4542 | | 7 | 1413 | 470 | 51832.0 | 9.32 | 11 |
| 137 | | *GmAAP33* | Glyma04g09140 | | | 1344 | | 6 | 648 | 215 | 24865.8 | 8.79 | 0 |
| 138 | | *GmAAP34* | Glyma04g09150 | | | 2170 | | 5 | 1335 | 444 | 48683.0 | 9.80 | 10 |
| 139 | | *GmAAP35* | Glyma06g09280 | | | 2372 | | 5 | 1263 | 420 | 46216.4 | 9.64 | 9 |
|  | | GAT group |  | | |  | |  |  |  |  |  |  |
| 140 | | *GmGAT1* | Glyma12g30570 | | | 4766 | | 6 | 1296 | 431 | 47528.7 | 8.73 | 8 |
| 141 | | *GmGAT2* | Glyma17g05360 | | | 4136 | | 6 | 1110 | 369 | 40911.0 | 8.29 | 7 |
| 142 | | *GmGAT3* | Glyma12g30560 | | | 5085 | | 7 | 1245 | 414 | 45655.7 | 7.86 | 7 |
| 143 | | *GmGAT4* | Glyma17g05370 | | | 5555 | | 8 | 1302 | 433 | 48701.3 | 8.19 | 8 |
| 144 | | *GmGAT5* | Glyma17g05380 | | | 4044 | | 5 | 930 | 309 | 33692.6 | 9.44 | 8 |
| 145 | | *GmGAT6* | Glyma12g30550 | | | 2764 | | 7 | 927 | 309 | 34635.4 | 8.32 | 5 |
| 146 | | *GmGAT7* | Glyma04g21700 | | | 747 | | 2 | 438 | 146 | 16078.3 | 5.73 | 3 |
| 147 | | *GmGAT8* | Glyma09g03150 | | | 648 | | 2 | 399 | 133 | 14311.1 | 8.18 | 2 |
| 148 | | *GmGAT9* | Glyma15g36870 | | | 744 | | 2 | 528 | 176 | 19234.3 | 7.76 | 4 |
| 149 | | *GmGAT10* | Glyma15g21800 | | | 1680 | | 6 | 813 | 270 | 30690.9 | 9.44 | 5 |
| 150 | | *GmGAT11* | Glyma12g15590 | | | 756 | | 3 | 561 | 187 | 20456.5 | 8.33 | 4 |
| 151 | | *GmGAT12* | Glyma12g08980 | | | 4039 | | 5 | 1137 | 378 | 41417.2 | 8.73 | 8 |
| 152 | | *GmGAT13* | Glyma11g19500 | | | 6991 | | 8 | 1266 | 421 | 46397.7 | 9.46 | 10 |
| 153 | | *GmGAT14* | Glyma20g33000 | | | 2983 | | 6 | 1392 | 463 | 50645.5 | 9.18 | 11 |
| 154 | | *GmGAT15* | Glyma10g34540 | | | 2922 | | 6 | 1392 | 463 | 50710.6 | 9.17 | 11 |
| 155 | | *GmGAT16* | Glyma01g43390 | | | 4869 | | 6 | 1326 | 441 | 48281.3 | 8.65 | 11 |
| 156 | | *GmGAT17* | Glyma05g37000 | | | 9021 | | 6 | 1338 | 445 | 48828.0 | 8.76 | 10 |
| 157 | | *GmGAT18* | Glyma10g03800 | | | 2783 | | 6 | 1071 | 356 | 39873.4 | 9.47 | 9 |
| 158 | | *GmGAT19* | Glyma02g15960 | | | 1278 | | 3 | 624 | 207 | 23173.3 | 9.97 | 3 |
|  | | ProT group |  | | |  | |  |  |  |  |  |  |
| 159 | | *GmProT1* | Glyma05g02790 | | | 3542 | | 8 | 1206 | 401 | 45630.9 | 9.98 | 10 |
| 160 | | *GmProT2* | Glyma17g13460 | | | 2684 | | 6 | 1278 | 425 | 48554.6 | 9.80 | 11 |
| 161 | | *GmProT3* | Glyma05g02780 | | | 2907 | | 6 | 1230 | 409 | 46656.9 | 9.72 | 10 |
| 162 | | *GmProT4* | Glyma18g03530 | | | 3125 | | 6 | 1332 | 443 | 48732.3 | 9.26 | 11 |
| 163 | | *GmProT5* | Glyma11g34780 | | | 2921 | | 6 | 1335 | 444 | 49090.8 | 9.39 | 11 |
| 164 | | *GmProT6* | Glyma14g06850 | | | 3990 | | 6 | 1308 | 435 | 47635.1 | 9.14 | 11 |
| 165 | | *GmProT7* | Glyma02g42050 | | | 3863 | | 6 | 1302 | 433 | 47266.7 | 8.26 | 11 |
|  | | LHT group |  | | |  | |  |  |  |  |  |  |
| 166 | | *GmLHT1* | Glyma01g36590 | | | 4985 | | 4 | 1629 | 542 | 59487.1 | 9.63 | 10 |
| 167 | | *GmLHT2* | Glyma11g08770 | | | 4483 | | 4 | 1632 | 543 | 59463.1 | 9.69 | 10 |
| 168 | | *GmLHT3* | Glyma06g02210 | | | 3708 | | 4 | 1377 | 458 | 50757.2 | 9.06 | 11 |
| 169 | | *GmLHT4* | Glyma04g02110 | | | 3973 | | 2 | 864 | 287 | 31489.2 | 8.12 | 6 |
| 170 | | *GmLHT5* | Glyma13g31880 | | | 4954 | | 5 | 1551 | 516 | 57090.6 | 9.50 | 10 |
| 171 | | *GmLHT6* | Glyma15g07440 | | | 4313 | | 4 | 1551 | 516 | 57036.6 | 9.50 | 10 |
| 172 | | *GmLHT7* | Glyma11g10280 | | | 4248 | | 5 | 1611 | 536 | 59806.6 | 8.43 | 9 |
| 173 | | *GmLHT8* | Glyma12g02580 | | | 3941 | | 5 | 1179 | 392 | 43147.6 | 9.87 | 7 |
| 174 | | *GmLHT9* | Glyma01g21510 | | | 4166 | | 7 | 1314 | 437 | 48910.0 | 9.12 | 9 |
| 175 | | *GmLHT10* | Glyma02g10870 | | | 8115 | | 8 | 1233 | 410 | 45880.1 | 9.45 | 10 |
| 176 | | *GmLHT11* | Glyma10g34790 | | | 2853 | | 7 | 1287 | 428 | 47877.0 | 9.12 | 9 |
| 177 | | *GmLHT12* | Glyma04g43450 | | | 2190 | | 6 | 1293 | 431 | 47973.0 | 9.54 | 8 |
| 178 | | *GmLHT13* | Glyma06g42970 | | | 943 | | 4 | 547 | 183 | 21278.4 | 8.73 | 4 |
| 179 | | *GmLHT14* | Glyma17g13710 | | | 2539 | | 6 | 1281 | 426 | 48139.2 | 8.29 | 11 |
| 180 | | *GmLHT15* | Glyma05g03060 | | | 1919 | | 2 | 906 | 302 | 33610.3 | 7.53 | 7 |
| 181 | | *GmLHT16* | Glyma18g01300 | | | 2682 | | 6 | 1302 | 433 | 47748.5 | 7.99 | 9 |
| 182 | | *GmLHT17* | Glyma11g37340 | | | 2917 | | 6 | 1290 | 429 | 47124.7 | 7.87 | 10 |
| 183 | | *GmLHT18* | Glyma08g10740 | | | 2162 | | 6 | 1275 | 424 | 47196.7 | 9.11 | 11 |
| 184 | | *GmLHT19* | Glyma05g27770 | | | 1682 | | 4 | 849 | 283 | 31163.7 | 7.57 | 7 |
| 185 | | *GmLHT20* | Glyma16g06740 | | | 5106 | | 6 | 1218 | 405 | 45326.6 | 9.17 | 8 |
| 186 | | *GmLHT21* | Glyma19g24540 | | | 4713 | | 7 | 1275 | 424 | 47840.7 | 8.60 | 7 |
| 187 | | *GmLHT22* | Glyma19g22590 | | | 9662 | | 7 | 1356 | 451 | 50827.9 | 8.98 | 11 |
| 188 | | *GmLHT23* | Glyma19g24520 | | | 2657 | | 6 | 1302 | 433 | 48605.7 | 9.33 | 11 |
| 189 | | *GmLHT24* | Glyma16g06750 | | | 3117 | | 6 | 1197 | 398 | 44578.0 | 9.25 | 10 |

*^a^* Systematic designation given to soybean *AATs* in this study.

*^b^* Locus identity number of *GmAATs* .

*^c^* Gene full length of *GmAATs*.

*^d^* Number of intron in *GmAAT* genes.

*^e^* Length of the open reading frame for *GmAATs*.

*^f^* Protein characterization of GmAATs.

*^g^* Number of transmembrane segments possessed by GmAATs, predicted by the TMHMM Server v2.0.
