# Supplementary material for: Genome-Wide Identification, Classification, and Expression Analysis of Amino Acid Transporter Gene Family in Glycine Max
Source: Front Plant Sci. 2016 Apr 20;7:515. doi: 10.3389/fpls.2016.00515 (PMC4837150; doi:10.3389/fpls.2016.00515)
Supplement: Table S2 — The expression data of GmAAT genes in various organs. [file Table2.DOCX]

| **Gene** | **Locus** | **Root** | **Hypocotyl** | **Cotyledon** | **Callus** | **SAM6D** | **SAM17D** | **SAM38D** | **Axillary** | **IBM** | **IAM** | **OF** |
| --- | --- | --- | --- | --- | --- | --- | --- | --- | --- | --- | --- | --- |
| GmCAT7 | Glyma05g30510 | 0.03 | 0.01 | 0.02 | 0.01 | 0.02 | 0.02 | 0.01 | 0.00 | 0.89 | 0.55 | 1.41 |
| GmCAT8 | Glyma08g13660 | 0.24 | 0.16 | 0.01 | 0.46 | 1.18 | 0.84 | 0.53 | 0.50 | 0.79 | 0.93 | 0.69 |
| GmCAT9 | Glyma08g13640 | 2.56 | 0.59 | 0.27 | 27.06 | 0.92 | 1.26 | 1.96 | 2.86 | 5.40 | 5.66 | 3.24 |
| GmCAT10 | Glyma09g10300 | 0.14 | 0.02 | 0.02 | 0.00 | 0.11 | 0.33 | 0.20 | 1.20 | 3.60 | 2.90 | 0.46 |
| GmCAT11 | Glyma09g21070 | 0.04 | 0.02 | 0.01 | 0.03 | 0.01 | 0.01 | 0.01 | 0.01 | 0.01 | 0.02 | 0.02 |
| GmCAT12 | Glyma09g01230 | 4.09 | 7.94 | 2.59 | 4.99 | 15.49 | 19.12 | 13.30 | 18.38 | 16.14 | 13.75 | 11.12 |
| GmCAT13 | Glyma09g05580 | 0 | 0.15 | 1.76 | 2.02 | 0.36 | 0.04 | 0.80 | 0.79 | 1.61 | 1.94 | 0.61 |
| GmCAT14 | Glyma09g05540 | 0.03 | 0.68 | 0.65 | 5.92 | 0.36 | 0.49 | 1.34 | 1.12 | 1.36 | 1.18 | 0.62 |
| GmCAT15 | Glyma17g15840 | 0.14 | 0.33 | 0.00 | 0.40 | 0.04 | 0.04 | 0.07 | 0.05 | 0.07 | 0.17 | 0.48 |
| GmCAT16 | Glyma05g05510 | 0.08 | 0.66 | 0.03 | 0.46 | 0.03 | 0.16 | 0.42 | 0.13 | 0.18 | 0.14 | 0.19 |
| GmCAT17 | Glyma11g04100 | 0.02 | 0.02 | 0.01 | 0.00 | 0.02 | 0.00 | 0.11 | 0.10 | 0.06 | 0.00 | 0.37 |
| GmCAT18 | Glyma20g24620 | 1.17 | 1.99 | 8.47 | 0.49 | 10.72 | 8.09 | 6.74 | 9.54 | 12.07 | 10.37 | 3.86 |
| GmCAT19 | Glyma10g42440 | 4.16 | 1.31 | 12.46 | 2.19 | 6.98 | 4.61 | 2.92 | 8.38 | 20.56 | 19.19 | 2.25 |
| GmCAT1 | Glyma09g37700 | 6.23 | 19.23 | 7.37 | 20.71 | 11.31 | 14.25 | 19.19 | 22.09 | 15.11 | 16.16 | 14.75 |
| GmCAT2 | Glyma18g48860 | 2.07 | 2.29 | 1.41 | 5.65 | 2.79 | 2.13 | 3.37 | 4.20 | 3.38 | 2.90 | 2.13 |
| GmCAT3 | Glyma16g04050 | 2.76 | 18.85 | 34.68 | 58.61 | 18.22 | 19.35 | 14.67 | 18.11 | 17.77 | 13.37 | 11.75 |
| GmCAT4 | Glyma19g29440 | 3.77 | 9.86 | 12.72 | 39.82 | 17.27 | 22.28 | 11.66 | 12.69 | 18.16 | 17.54 | 13.52 |
| GmCAT5 | Glyma05g11410 | 2.69 | 10.97 | 3.06 | 4.97 | 2.45 | 2.55 | 3.57 | 5.47 | 4.97 | 5.75 | 4.81 |
| GmCAT6 | Glyma19g29450 | 9.05 | 5.51 | 0.02 | 0.21 | 0.09 | 0.01 | 0.21 | 0.19 | 0.42 | 0.69 | 1.42 |
| GmPHS1 | Glyma14g35520 | 0.22 | 0.43 | 0.29 | 0.16 | 1.94 | 2.99 | 2.04 | 2.15 | 1.59 | 0.79 | 1.19 |
| GmPHS2 | Glyma02g37200 | 3.18 | 6.98 | 5.73 | 12.27 | 5.10 | 11.98 | 16.66 | 18.24 | 21.67 | 26.56 | 7.86 |
| GmPHS3 | Glyma05g28810 | 0.68 | 3.19 | 0.69 | 0.63 | 2.19 | 2.39 | 2.48 | 3.49 | 3.65 | 4.15 | 20.03 |
| GmPHS4 | Glyma08g11960 | 1.10 | 10.32 | 2.42 | 1.22 | 4.40 | 2.93 | 4.29 | 4.81 | 6.72 | 8.17 | 32.99 |
| GmPHS5 | Glyma01g45410 | 3.98 | 4.84 | 12.63 | 3.94 | 3.73 | 3.33 | 3.46 | 5.53 | 3.66 | 5.18 | 2.59 |
| GmPHS6 | Glyma20g24020 | 0.05 | 0.04 | 0.01 | 0.03 | 0.00 | 0.01 | 0.07 | 0.01 | 0.11 | 0.00 | 0.02 |
| GmPHS7 | Glyma16g17460 | 0.04 | 0.10 | 1.19 | 0.00 | 0.91 | 2.20 | 0.94 | 0.61 | 0.38 | 0.15 | 0.05 |
| GmPHS8 | Glyma01g35320 | 9.47 | 18.83 | 12.74 | 7.28 | 15.53 | 13.44 | 13.94 | 19.02 | 33.66 | 25.06 | 16.33 |
| GmPHS9 | Glyma09g34750 | 19.20 | 37.71 | 12.26 | 11.99 | 18.06 | 20.58 | 21.74 | 23.90 | 22.93 | 21.36 | 22.34 |
| GmACT1 | Glyma10g41570 | 0.09 | 0.00 | 0.00 | 0.00 | 0.03 | 0.00 | 0.00 | 0.00 | 0.12 | 0.03 | 0.00 |
| GmACT2 | Glyma04g38710 | 4.82 | 0.69 | 0.18 | 8.22 | 3.53 | 2.81 | 2.26 | 2.54 | 5.34 | 2.89 | 1.15 |
| GmACT3 | Glyma06g16280 | 7.83 | 0.28 | 0.03 | 0.77 | 0.78 | 0.51 | 0.55 | 0.49 | 1.84 | 0.86 | 0.27 |
| GmACT4 | Glyma20g25660 | 2.53 | 2.32 | 2.48 | 4.83 | 1.46 | 1.20 | 1.11 | 1.63 | 1.73 | 2.23 | 1.64 |
| GmACT5 | Glyma20g25680 | 4.39 | 2.93 | 4.68 | 0.33 | 2.77 | 2.66 | 2.14 | 3.00 | 5.38 | 10.09 | 4.87 |
| GmACT6 | Glyma10g41560 | 12.88 | 11.22 | 16.77 | 5.27 | 10.85 | 7.63 | 5.90 | 6.21 | 11.03 | 10.51 | 4.71 |
| GmACT7 | Glyma20g25670 | 9.75 | 5.71 | 5.53 | 0.68 | 2.84 | 2.16 | 1.61 | 1.41 | 1.27 | 1.26 | 0.64 |
| GmTTP1 | Glyma16g27100 | 0.17 | 5.65 | 22.04 | 0.23 | 5.12 | 3.85 | 3.31 | 5.04 | 6.75 | 5.90 | 1.02 |
| GmATL1 | Glyma15g00870 | 0.95 | 1.15 | 0.27 | 0.91 | 0.87 | 0.38 | 0.97 | 1.44 | 1.73 | 1.65 | 2.14 |
| GmATL2 | Glyma13g44450 | 4.02 | 3.08 | 0.44 | 2.74 | 2.36 | 1.94 | 2.69 | 3.43 | 2.77 | 1.96 | 4.32 |
| GmATL3 | Glyma19g05000 | 1.00 | 5.91 | 3.78 | 12.51 | 2.09 | 2.45 | 1.94 | 3.39 | 4.69 | 3.89 | 7.74 |
| GmATL4 | Glyma13g06930 | 4.29 | 6.52 | 8.24 | 29.09 | 5.71 | 5.10 | 7.50 | 8.02 | 8.03 | 5.09 | 8.17 |
| GmATL5 | Glyma19g04990 | 2.85 | 7.42 | 1.82 | 6.16 | 3.94 | 5.32 | 9.26 | 10.01 | 10.08 | 8.93 | 4.60 |
| GmATL6 | Glyma08g28190 | 23.17 | 12.92 | 11.43 | 95.44 | 21.34 | 13.30 | 20.47 | 31.61 | 30.78 | 35.12 | 132.17 |
| GmATL7 | Glyma18g51220 | 5.02 | 12.25 | 10.82 | 149.73 | 12.63 | 8.22 | 14.02 | 16.37 | 17.85 | 20.64 | 55.89 |
| GmATL8 | Glyma06g29640 | 11.69 | 8.48 | 4.48 | 7.18 | 3.02 | 3.78 | 3.45 | 4.12 | 3.44 | 3.55 | 6.18 |
| GmATL9 | Glyma14g10260 | 1.24 | 0.57 | 1.66 | 0.02 | 4.78 | 2.22 | 1.78 | 1.15 | 1.80 | 1.01 | 3.78 |
| GmATL10 | Glyma14g05910 | 26.04 | 34.19 | 12.26 | 9.10 | 8.12 | 13.39 | 7.05 | 8.84 | 7.94 | 9.23 | 8.56 |
| GmATL11 | Glyma02g42810 | 16.23 | 39.80 | 20.84 | 3.23 | 17.86 | 25.60 | 22.29 | 24.01 | 17.35 | 22.43 | 16.00 |
| GmATL12 | Glyma14g05900 | 0.12 | 0.11 | 0.13 | 0.00 | 0.00 | 0.03 | 0.00 | 0.00 | 0.03 | 0.09 | 0.39 |
| GmATL13 | Glyma02g42800 | 4.04 | 29.33 | 15.47 | 29.15 | 8.73 | 9.34 | 15.79 | 13.70 | 4.38 | 8.87 | 17.14 |
| GmATL14 | Glyma14g05890 | 2.32 | 40.40 | 7.54 | 17.56 | 3.03 | 4.22 | 2.29 | 3.10 | 1.23 | 1.59 | 9.82 |
| GmATL15 | Glyma18g02580 | 2.28 | 21.15 | 15.81 | 58.65 | 8.64 | 7.74 | 8.55 | 9.59 | 9.80 | 8.88 | 34.71 |
| GmATL16 | Glyma11g35830 | 2.82 | 26.36 | 27.86 | 67.29 | 12.46 | 26.33 | 27.03 | 21.87 | 19.31 | 23.76 | 47.47 |
| GmANT1 | Glyma09g33430 | 0.06 | 0.63 | 0.06 | 0.82 | 0.49 | 0.40 | 0.73 | 0.91 | 0.50 | 0.66 | 0.62 |
| GmANT2 | Glyma11g09190 | 4.23 | 12.51 | 18.91 | 34.95 | 9.49 | 12.73 | 15.49 | 13.89 | 9.52 | 5.47 | 31.43 |
| GmANT3 | Glyma01g36250 | 2.11 | 11.81 | 0.97 | 9.14 | 6.10 | 11.37 | 9.89 | 8.02 | 6.96 | 8.73 | 18.58 |
| GmANT4 | Glyma09g33030 | 0.03 | 0.02 | 0.02 | 0.01 | 0.03 | 0.01 | 0.05 | 0.00 | 0.04 | 0.09 | 12.18 |
| GmANT5 | Glyma19g39060 | 6.51 | 5.99 | 3.33 | 10.22 | 5.11 | 2.80 | 4.51 | 5.16 | 5.02 | 4.07 | 8.37 |
| GmANT6 | Glyma03g36410 | 4.00457 | 12.2879 | 11.9773 | 4.43473 | 7.24362 | 6.97888 | 5.26275 | 7.74236 | 4.59296 | 6.18691 | 6.02219 |
| GmATL17 | Glyma11g36880 | 0.01 | 0.03 | 0.01 | 0.03 | 0.00 | 0.01 | 0.00 | 0.93 | 0.50 | 0.79 | 1.49 |
| GmATL18 | Glyma18g00780 | 0.01 | 0.02 | 0.01 | 0.00 | 0.00 | 0.01 | 0.04 | 0.00 | 0.47 | 0.49 | 0.47 |
| GmATL19 | Glyma05g28160 | 0.01 | 0.01 | 0.05 | 0.01 | 0.01 | 0.01 | 0.00 | 0.00 | 0.00 | 0.01 | 0.01 |
| GmATL20 | Glyma02g30960 | 5.74 | 23.98 | 9.40 | 0.87 | 13.87 | 19.55 | 18.58 | 16.24 | 19.28 | 18.44 | 8.29 |
| GmATL21 | Glyma10g12290 | 2.04 | 14.71 | 6.27 | 0.00 | 1.32 | 1.62 | 2.09 | 2.06 | 1.44 | 1.43 | 2.07 |
| GmATL22 | Glyma19g31090 | 0.04 | 0.00 | 0.00 | 0.01 | 0.00 | 0.01 | 0.01 | 0.00 | 1.36 | 2.07 | 0.38 |
| GmATL23 | Glyma03g28370 | 0.01 | 0.00 | 0.01 | 0.00 | 0.14 | 0.33 | 0.27 | 0.45 | 1.08 | 1.33 | 0.56 |
| GmATL24 | Glyma14g15070 | 0.61 | 0.40 | 0.71 | 0.33 | 0.37 | 0.13 | 0.17 | 1.65 | 47.21 | 64.24 | 4.28 |
| GmATL25 | Glyma01g42750 | 10.56 | 1.51 | 10.10 | 9.53 | 2.90 | 0.80 | 3.42 | 3.28 | 8.36 | 7.45 | 3.12 |
| GmATL26 | Glyma11g02700 | 0.12 | 0.06 | 0.06 | 0.06 | 0.14 | 0.00 | 0.26 | 0.00 | 0.41 | 0.06 | 0.04 |
| GmATL27 | Glyma09g24210 | 0.21 | 0.05 | 0.11 | 0.01 | 0.21 | 0.18 | 0.03 | 0.08 | 0.72 | 1.01 | 0.09 |
| GmATL28 | Glyma01g27180 | 0.01 | 0.11 | 0.01 | 0.00 | 0.02 | 0.04 | 0.05 | 0.02 | 0.19 | 0.02 | 0.00 |
| GmATL29 | Glyma18g49420 | 0.01 | 0.71 | 0.28 | 0.07 | 0.15 | 0.25 | 0.09 | 0.21 | 0.57 | 0.60 | 0.01 |
| GmATL30 | Glyma09g39320 | 0.60 | 0.06 | 0.01 | 0.03 | 0.12 | 0.45 | 0.04 | 0.22 | 0.64 | 0.55 | 0.56 |
| GmATL31 | Glyma09g37270 | 3.43 | 9.92 | 0.50 | 30.35 | 9.01 | 28.16 | 7.40 | 12.62 | 20.16 | 16.42 | 6.44 |
| GmATL32 | Glyma09g37260 | 0.01 | 0.05 | 0.02 | 0.13 | 0.38 | 0.56 | 0.70 | 0.29 | 0.79 | 0.51 | 14.34 |
| GmATL33 | Glyma18g11330 | 0.00 | 0.01 | 0.00 | 0.00 | 0.01 | 0.00 | 0.00 | 0.03 | 0.02 | 0.03 | 0.24 |
| GmATL34 | Glyma11g29030 | 0.02 | 0.07 | 0.04 | 0.00 | 0.02 | 0.41 | 0.04 | 0.21 | 0.00 | 0.00 | 0.00 |
| GmATL35 | Glyma18g06650 | 11.66 | 7.70 | 3.92 | 0.75 | 11.74 | 16.58 | 15.49 | 14.92 | 8.87 | 8.62 | 3.27 |
| GmATL36 | Glyma11g29080 | 0.69 | 0.47 | 0.09 | 0.19 | 2.25 | 3.32 | 4.10 | 2.98 | 1.31 | 0.98 | 25.62 |
| GmATL37 | Glyma11g29050 | 1.85 | 0.88 | 0.47 | 0.87 | 11.06 | 11.70 | 12.33 | 13.01 | 5.20 | 5.83 | 71.95 |
| GmATL38 | Glyma20g04840 | 0.01 | 0.01 | 0.32 | 0.07 | 0.00 | 0.02 | 0.22 | 0.62 | 2.10 | 4.13 | 23.35 |
| GmATL39 | Glyma1675s00200 | 0.00 | 0.00 | 0.04 | 0.03 | 0.03 | 0.00 | 0.03 | 0.00 | 0.03 | 0.00 | 0.00 |
| GmATL40 | Glyma18g40080 | 0.02 | 0.00 | 0.01 | 0.00 | 0.00 | 0.00 | 0.02 | 0.00 | 0.00 | 0.00 | 0.00 |
| GmATL41 | Glyma02g19430 | 38.05 | 2.87 | 1.39 | 1.72 | 1.11 | 0.23 | 0.93 | 1.09 | 2.14 | 2.39 | 4.96 |
| GmATL42 | Glyma10g15130 | 33.93 | 6.76 | 1.56 | 6.09 | 1.13 | 0.31 | 1.14 | 2.11 | 1.31 | 5.33 | 9.96 |
| GmATL43 | Glyma20g32260 | 7.23 | 61.71 | 22.84 | 14.64 | 8.40 | 7.61 | 5.86 | 9.08 | 8.49 | 11.79 | 20.17 |
| GmATL44 | Glyma10g35280 | 52.81 | 68.40 | 26.62 | 38.26 | 8.14 | 4.36 | 7.43 | 14.53 | 19.12 | 29.67 | 36.17 |
| GmATL45 | Glyma09g26880 | 1.34 | 8.02 | 0.60 | 0.49 | 0.96 | 0.46 | 0.63 | 1.20 | 1.29 | 2.39 | 2.64 |
| GmATL46 | Glyma16g08770 | 3.74 | 27.24 | 8.43 | 7.72 | 5.55 | 6.05 | 3.87 | 4.14 | 4.99 | 8.39 | 10.70 |
| GmAUX1 | Glyma06g00690 | 0.44 | 1.10 | 0.09 | 0.01 | 3.09 | 1.16 | 1.17 | 1.21 | 1.33 | 0.65 | 0.57 |
| GmAUX2 | Glyma04g00640 | 1.09 | 2.31 | 0.03 | 0.00 | 5.23 | 1.24 | 1.30 | 1.74 | 1.99 | 1.77 | 0.43 |
| GmAUX3 | Glyma11g11310 | 12.03 | 7.70 | 3.09 | 0.78 | 22.16 | 22.90 | 12.59 | 13.76 | 14.90 | 5.93 | 2.96 |
| GmAUX4 | Glyma12g03490 | 4.71 | 2.66 | 2.21 | 0.58 | 15.15 | 14.02 | 10.93 | 6.95 | 9.49 | 2.85 | 1.14 |
| GmAUX5 | Glyma06g11540 | 33.91 | 19.59 | 0.75 | 1.03 | 4.21 | 6.04 | 9.58 | 12.56 | 6.30 | 8.55 | 27.49 |
| GmAUX6 | Glyma04g43150 | 66.37 | 24.35 | 2.18 | 3.70 | 3.22 | 5.71 | 8.48 | 11.47 | 6.22 | 11.43 | 16.35 |
| GmAUX7 | Glyma11g35080 | 0.88 | 5.89 | 1.30 | 2.09 | 7.95 | 7.16 | 6.16 | 4.04 | 4.04 | 1.95 | 3.57 |
| GmAUX8 | Glyma18g03280 | 0.12 | 0.48 | 0.95 | 0.68 | 2.31 | 1.56 | 1.06 | 0.53 | 0.37 | 0.18 | 1.23 |
| GmAUX9 | Glyma14g06600 | 0.12 | 2.40 | 0.04 | 10.82 | 0.94 | 0.21 | 0.37 | 0.97 | 3.17 | 3.74 | 21.94 |
| GmAUX10 | Glyma02g42290 | 0.09 | 1.69 | 0.02 | 3.35 | 0.67 | 0.53 | 0.74 | 1.57 | 5.01 | 4.23 | 7.31 |
| GmAUX11 | Glyma07g17810 | 18.00 | 32.55 | 1.79 | 8.74 | 3.11 | 5.31 | 4.40 | 9.12 | 12.96 | 13.54 | 71.00 |
| GmAUX12 | Glyma18g42640 | 10.81 | 9.39 | 1.23 | 9.32 | 2.22 | 3.34 | 3.84 | 6.95 | 7.31 | 8.31 | 17.03 |
| GmAUX13 | Glyma01g28060 | 13.17 | 13.81 | 0.27 | 0.00 | 0.03 | 0.00 | 0.06 | 0.31 | 0.37 | 0.28 | 0.00 |
| GmAUX14 | Glyma03g09140 | 52.24 | 33.52 | 4.52 | 0.10 | 0.51 | 0.10 | 0.07 | 0.12 | 0.48 | 0.40 | 0.54 |
| GmAUX15 | Glyma03g09100 | 37.49 | 35.74 | 14.14 | 1.31 | 6.96 | 14.16 | 5.77 | 7.54 | 11.18 | 18.44 | 11.44 |
| GmAUX16 | Glyma01g28310 | 18.08 | 16.99 | 14.29 | 2.22 | 2.66 | 9.79 | 4.14 | 3.19 | 4.42 | 9.13 | 17.57 |
| GmAAP1 | Glyma06g16340 | 3.00 | 11.17 | 10.84 | 11.61 | 5.52 | 3.17 | 4.47 | 6.66 | 9.44 | 18.86 | 34.03 |
| GmAAP2 | Glyma04g38650 | 4.31 | 3.46 | 4.40 | 3.32 | 4.53 | 3.80 | 5.04 | 5.66 | 13.04 | 14.93 | 48.41 |
| GmAAP3 | Glyma04g38640 | 8.65 | 23.82 | 38.03 | 85.46 | 22.37 | 25.24 | 26.89 | 33.95 | 39.14 | 33.62 | 24.65 |
| GmAAP4 | Glyma06g16350 | 8.20 | 8.42 | 13.94 | 10.00 | 5.51 | 7.11 | 7.76 | 5.76 | 6.05 | 6.16 | 7.04 |
| GmAAP5 | Glyma05g32810 | 2.41 | 6.49 | 0.04 | 0.29 | 0.03 | 0.06 | 0.36 | 0.19 | 0.21 | 0.22 | 0.03 |
| GmAAP6 | Glyma08g00460 | 0.01 | 0.10 | 0.01 | 0.00 | 0.00 | 0.04 | 0.00 | 0.06 | 0.00 | 0.00 | 0.05 |
| GmAAP7 | Glyma13g10070 | 0.04 | 4.03 | 4.21 | 1.12 | 1.30 | 7.43 | 3.47 | 8.09 | 19.09 | 21.74 | 26.91 |
| GmAAP8 | Glyma14g24370 | 0.06 | 1.43 | 3.59 | 0.62 | 0.25 | 0.13 | 0.81 | 0.91 | 3.17 | 2.25 | 42.01 |
| GmAAP9 | Glyma04g42520 | 0.08 | 4.17 | 4.98 | 0.00 | 0.13 | 0.11 | 0.42 | 2.72 | 2.65 | 4.51 | 3.37 |
| GmAAP10 | Glyma06g12270 | 0.07 | 8.61 | 1.03 | 0.42 | 0.07 | 0.01 | 0.46 | 1.34 | 3.06 | 2.51 | 0.39 |
| GmAAP11 | Glyma02g34510 | 0.02 | 0.88 | 0.44 | 0.19 | 0.42 | 0.46 | 0.74 | 0.97 | 1.11 | 0.39 | 1.46 |
| GmAAP12 | Glyma04g32730 | 0.00 | 0.18 | 0.27 | 0.00 | 0.00 | 0.00 | 0.08 | 0.07 | 0.08 | 0.00 | 0.45 |
| GmAAP13 | Glyma11g11440 | 0.02 | 5.80 | 0.70 | 0.05 | 0.03 | 0.21 | 0.32 | 0.72 | 0.41 | 0.57 | 2.13 |
| GmAAP14 | Glyma12g03580 | 0.07 | 5.50 | 0.10 | 0.33 | 0.59 | 0.77 | 1.06 | 2.16 | 0.74 | 0.39 | 0.58 |
| GmAAP15 | Glyma08g44940 | 1.61 | 1.13 | 2.62 | 0.11 | 0.53 | 0.02 | 0.36 | 0.90 | 1.24 | 1.51 | 1.73 |
| GmAAP16 | Glyma18g07970 | 3.48 | 1.46 | 9.96 | 1.30 | 0.36 | 0.69 | 0.60 | 0.41 | 1.89 | 1.56 | 4.12 |
| GmAAP17 | Glyma02g47370 | 17.01 | 20.20 | 10.32 | 20.60 | 7.72 | 7.17 | 9.00 | 9.29 | 8.67 | 10.84 | 5.47 |
| GmAAP18 | Glyma14g01370 | 1.60 | 4.68 | 3.56 | 10.97 | 1.29 | 0.72 | 1.52 | 0.95 | 1.81 | 1.22 | 0.71 |
| GmAAP19 | Glyma02g47350 | 0.54 | 4.13 | 5.48 | 29.51 | 8.87 | 14.57 | 15.01 | 10.89 | 13.66 | 14.10 | 17.87 |
| GmAAP20 | Glyma14g01410 | 0.42 | 0.40 | 0.72 | 12.26 | 2.97 | 5.17 | 4.80 | 3.71 | 3.65 | 1.78 | 2.72 |
| GmAAP21 | Glyma18g07980 | 0.01 | 0.17 | 0.45 | 0.06 | 0.07 | 0.06 | 0.08 | 0.26 | 0.36 | 0.66 | 0.23 |
| GmAAP22 | Glyma17g32240 | 0.29 | 3.00 | 2.26 | 1.70 | 0.59 | 0.43 | 1.29 | 0.67 | 0.87 | 0.78 | 2.74 |
| GmAAP23 | Glyma19g07580 | 0.03 | 0.28 | 0.01 | 0.05 | 0.03 | 0.18 | 0.42 | 0.12 | 0.08 | 0.09 | 0.51 |
| GmAAP24 | Glyma18g08000 | 1.12 | 3.37 | 4.54 | 5.03 | 3.36 | 2.37 | 3.99 | 5.36 | 3.38 | 5.87 | 20.13 |
| GmAAP25 | Glyma08g44930 | 2.53 | 49.54 | 8.10 | 37.38 | 18.01 | 24.90 | 20.45 | 26.08 | 21.13 | 17.49 | 79.59 |
| GmAAP26 | Glyma10g40130 | 0.02 | 0.01 | 0.02 | 0.00 | 0.05 | 0.00 | 0.08 | 0.01 | 0.29 | 0.00 | 0.01 |
| GmAAP27 | Glyma17g26590 | 0.15 | 13.15 | 69.74 | 5.11 | 16.92 | 40.06 | 32.51 | 53.15 | 64.39 | 61.67 | 189.69 |
| GmAAP28 | Glyma06g09470 | 0.11 | 7.98 | 2.50 | 0.70 | 0.35 | 0.41 | 1.90 | 4.09 | 5.70 | 5.78 | 7.62 |
| GmAAP29 | Glyma04g09310 | 0.25 | 28.91 | 10.04 | 1.22 | 0.51 | 0.60 | 3.06 | 6.85 | 8.91 | 10.61 | 8.44 |
| GmAAP30 | Glyma14g22120 | 7.02 | 11.29 | 24.67 | 15.49 | 19.37 | 8.72 | 16.16 | 18.15 | 16.91 | 17.62 | 13.82 |
| GmAAP31 | Glyma14g21870 | 0.00 | 0.00 | 0.00 | 0.06 | 0.00 | 0.00 | 0.03 | 0.00 | 0.03 | 0.00 | 0.00 |
| GmAAP32 | Glyma06g09270 | 0.07 | 3.31 | 2.04 | 2.04 | 1.17 | 4.42 | 3.23 | 6.21 | 4.99 | 5.11 | 3.80 |
| GmAAP33 | Glyma04g09140 | 0.01 | 0.20 | 0.01 | 0.02 | 0.02 | 0.02 | 0.12 | 0.31 | 0.37 | 0.02 | 0.22 |
| GmAAP34 | Glyma04g09150 | 15.14 | 3.64 | 4.64 | 0.42 | 0.97 | 1.26 | 2.08 | 3.63 | 2.97 | 6.30 | 8.67 |
| GmAAP35 | Glyma06g09280 | 2.10 | 8.46 | 18.62 | 0.53 | 3.96 | 3.09 | 4.64 | 9.74 | 5.29 | 8.11 | 10.91 |
| GmGAT1 | Glyma12g30570 | 0.03 | 0.57 | 0.01 | 0.01 | 0.09 | 0.02 | 0.11 | 0.04 | 0.52 | 0.39 | 0.21 |
| GmGAT2 | Glyma17g05360 | 0.06 | 2.34 | 0.84 | 0.00 | 0.05 | 0.03 | 0.12 | 0.05 | 0.18 | 0.08 | 0.42 |
| GmGAT3 | Glyma12g30560 | 0.04 | 0.13 | 0.00 | 0.00 | 0.44 | 0.10 | 0.43 | 0.12 | 0.05 | 0.01 | 0.06 |
| GmGAT4 | Glyma17g05370 | 0.02 | 0.05 | 0.015 | 0.00 | 0.12 | 0.00 | 0.05 | 0.25 | 0.06 | 0.00 | 0.20 |
| GmGAT5 | Glyma17g05380 | 0.04 | 0.03 | 0.07 | 1.96 | 0.17 | 0.07 | 0.13 | 1.93 | 25.38 | 28.43 | 1.00 |
| GmGAT6 | Glyma12g30550 | 0.03 | 0.02 | 0.00 | 0.18 | 0.06 | 0.02 | 0.00 | 0.11 | 1.65 | 2.24 | 0.39 |
| GmGAT7 | Glyma04g21700 | 0.00 | 0.02 | 0.00 | 0.00 | 0.00 | 0.00 | 0.04 | 0.00 | 0.00 | 0.00 | 0.00 |
| GmGAT8 | Glyma09g03150 | 0.00 | 0.00 | 0.00 | 0.00 | 0.04 | 0.00 | 0.04 | 0.00 | 0.00 | 0.00 | 0.00 |
| GmGAT9 | Glyma15g36870 | 0.02 | 0.01 | 0.00 | 0.00 | 0.00 | 0.00 | 0.00 | 0.00 | 0.00 | 0.00 | 0.00 |
| GmGAT10 | Glyma15g21800 | 0.01 | 0.01 | 0.01 | 0.00 | 0.05 | 0.04 | 0.02 | 0.00 | 0.00 | 0.02 | 0.00 |
| GmGAT11 | Glyma12g15590 | 0.00 | 0.03 | 0.00 | 0.00 | 0.00 | 0.00 | 0.00 | 0.03 | 0.00 | 0.03 | 0.00 |
| GmGAT12 | Glyma12g08980 | 2.50 | 1.32 | 1.41 | 24.67 | 4.86 | 7.99 | 7.45 | 7.75 | 12.64 | 13.43 | 12.52 |
| GmGAT13 | Glyma11g19500 | 0.68 | 0.51 | 3.03 | 6.07 | 1.52 | 2.18 | 3.01 | 1.73 | 3.92 | 6.16 | 3.24 |
| GmGAT14 | Glyma20g33000 | 3.86 | 0.47 | 0.03 | 2.62 | 0.49 | 0.46 | 0.91 | 0.91 | 0.59 | 0.38 | 1.63 |
| GmGAT15 | Glyma10g34540 | 14.85 | 1.92 | 0.55 | 6.11 | 1.51 | 1.52 | 3.36 | 2.31 | 1.97 | 1.76 | 3.07 |
| GmGAT16 | Glyma01g43390 | 0.82 | 1.07 | 0.56 | 0.01 | 3.02 | 2.21 | 5.42 | 4.43 | 2.76 | 0.84 | 1.56 |
| GmGAT17 | Glyma05g37000 | 0.78 | 2.09 | 4.01 | 54.20 | 2.84 | 2.63 | 8.80 | 12.54 | 11.72 | 22.85 | 50.92 |
| GmGAT18 | Glyma10g03800 | 0.05 | 0.17 | 0.00 | 2.25 | 0.48 | 0.43 | 0.84 | 0.79 | 0.58 | 1.02 | 3.05 |
| GmGAT19 | Glyma02g15960 | 0.05 | 0.01 | 0.03 | 0.35 | 0.05 | 0.24 | 0.16 | 0.16 | 0.15 | 0.02 | 0.07 |
| GmProT1 | Glyma05g02790 | 0.19 | 1.61 | 2.92 | 0.66 | 0.03 | 0.28 | 1.70 | 1.92 | 2.93 | 2.23 | 0.72 |
| GmProT2 | Glyma17g13460 | 0.01 | 0.02 | 0.46 | 0.21 | 0.00 | 0.00 | 0.01 | 0.15 | 0.08 | 0.03 | 0.00 |
| GmProT3 | Glyma05g02780 | 0.02 | 0.19 | 0.05 | 0.03 | 0.01 | 0.00 | 0.22 | 0.26 | 0.17 | 0.32 | 0.15 |
| GmProT4 | Glyma18g03530 | 0.12 | 16.56 | 41.75 | 5.45 | 2.14 | 0.69 | 3.28 | 5.22 | 8.95 | 11.03 | 34.61 |
| GmProT5 | Glyma11g34780 | 0.73 | 5.61 | 16.13 | 1.06 | 10.53 | 8.10 | 7.57 | 14.17 | 31.45 | 50.04 | 123.15 |
| GmProT6 | Glyma14g06850 | 0.63 | 1.91 | 0.66 | 0.79 | 2.44 | 2.16 | 4.96 | 4.68 | 5.02 | 4.14 | 27.03 |
| GmProT7 | Glyma02g42050 | 13.45 | 12.21 | 12.24 | 10.70 | 15.30 | 20.46 | 15.74 | 13.91 | 25.04 | 25.66 | 20.51 |
| GmLHT1 | Glyma01g36590 | 9.53 | 5.29 | 0.46 | 73.34 | 0.21 | 0.77 | 3.52 | 5.43 | 4.69 | 5.99 | 4.57 |
| GmLHT2 | Glyma11g08770 | 2.15 | 0.56 | 0.23 | 30.27 | 0.64 | 2.24 | 4.13 | 6.66 | 4.22 | 4.90 | 5.16 |
| GmLHT3 | Glyma06g02210 | 0.02 | 0.05 | 0.01 | 0.06 | 0.18 | 0.76 | 0.81 | 0.99 | 1.49 | 2.96 | 1.27 |
| GmLHT4 | Glyma04g02110 | 0.05 | 0.10 | 0.02 | 0.00 | 3.18 | 3.72 | 6.66 | 6.73 | 5.03 | 8.61 | 3.82 |
| GmLHT5 | Glyma13g31880 | 6.09 | 5.08 | 1.50 | 4.01 | 11.50 | 32.25 | 18.96 | 15.80 | 13.83 | 11.05 | 10.09 |
| GmLHT6 | Glyma15g07440 | 5.36 | 6.83 | 3.20 | 2.80 | 10.30 | 28.97 | 19.17 | 18.09 | 15.09 | 12.19 | 10.94 |
| GmLHT7 | Glyma11g10280 | 2.23 | 6.11 | 4.49 | 0.49 | 0.70 | 1.65 | 1.45 | 5.39 | 5.31 | 7.99 | 7.60 |
| GmLHT8 | Glyma12g02580 | 0.01 | 0.23 | 0.01 | 0.00 | 0.04 | 0.14 | 0.18 | 0.38 | 0.24 | 0.03 | 0.10 |
| GmLHT9 | Glyma01g21510 | 0.19 | 1.76 | 0.02 | 0.46 | 0.05 | 0.06 | 1.18 | 0.65 | 0.84 | 0.62 | 0.43 |
| GmLHT10 | Glyma02g10870 | 0.01 | 0.02 | 0.01 | 0.00 | 0.00 | 0.00 | 0.29 | 0.27 | 0.45 | 0.24 | 0.00 |
| GmLHT11 | Glyma10g34790 | 0.03 | 0.05 | 0.02 | 0.00 | 0.03 | 0.09 | 0.31 | 0.47 | 0.14 | 0.00 | 0.03 |
| GmLHT12 | Glyma04g43450 | 0.02 | 0.02 | 0.00 | 0.00 | 0.00 | 0.00 | 0.02 | 0.13 | 8.23 | 9.21 | 0.75 |
| GmLHT13 | Glyma06g42970 | 0.02 | 0.00 | 0.00 | 0.03 | 0.00 | 0.03 | 0.00 | 0.03 | 0.00 | 0.00 | 0.00 |
| GmLHT14 | Glyma17g13710 | 0.03 | 0.01 | 0.03 | 0.02 | 0.01 | 0.00 | 0.03 | 0.95 | 8.72 | 8.54 | 0.00 |
| GmLHT15 | Glyma05g03060 | 0.01 | 0.03 | 0.02 | 0.02 | 0.00 | 0.00 | 0.00 | 0.50 | 0.60 | 0.31 | 0.00 |
| GmLHT16 | Glyma18g01300 | 0.02 | 0.01 | 0.00 | 0.00 | 0.08 | 0.01 | 0.36 | 0.00 | 0.73 | 2.84 | 136.68 |
| GmLHT17 | Glyma11g37340 | 0.01 | 0.02 | 0.00 | 0.00 | 0.26 | 0.02 | 0.15 | 0.09 | 0.70 | 4.25 | 74.92 |
| GmLHT18 | Glyma08g10740 | 0.02 | 0.01 | 0.02 | 0.24 | 0.03 | 0.01 | 0.04 | 0.13 | 0.22 | 0.02 | 0.99 |
| GmLHT19 | Glyma05g27770 | 0.01 | 0.01 | 0.02 | 0.00 | 0.00 | 0.02 | 0.17 | 0.00 | 0.00 | 0.00 | 0.03 |
| GmLHT20 | Glyma16g06740 | 0.09 | 0.12 | 0.06 | 21.00 | 0.18 | 0.31 | 1.62 | 1.90 | 2.77 | 2.44 | 1.33 |
| GmLHT21 | Glyma19g24540 | 0.02 | 0.01 | 0.03 | 0.17 | 0.08 | 0.00 | 0.10 | 0.28 | 0.49 | 0.27 | 0.30 |
| GmLHT22 | Glyma19g22590 | 0.07 | 0.02 | 0.69 | 108.17 | 0.21 | 0.19 | 0.20 | 0.40 | 1.34 | 2.00 | 3.82 |
| GmLHT23 | Glyma19g24520 | 0.85 | 0.01 | 0.43 | 6.89 | 0.10 | 0.09 | 0.35 | 0.88 | 1.39 | 0.67 | 1.69 |
| GmLHT24 | Glyma16g06750 | 34.48 | 1.34 | 2.76 | 79.94 | 2.83 | 2.12 | 8.13 | 9.76 | 16.61 | 26.17 | 34.51 |
|  |  |  |  |  |  |  |  |  |  |  |  |  |
