# Supplementary figures and images for: Genome-Wide Identification, Classification, and Expression Analysis of Amino Acid Transporter Gene Family in Glycine Max
Source: Front Plant Sci. 2016 Apr 20;7:515. doi: 10.3389/fpls.2016.00515 (PMC4837150; doi:10.3389/fpls.2016.00515)

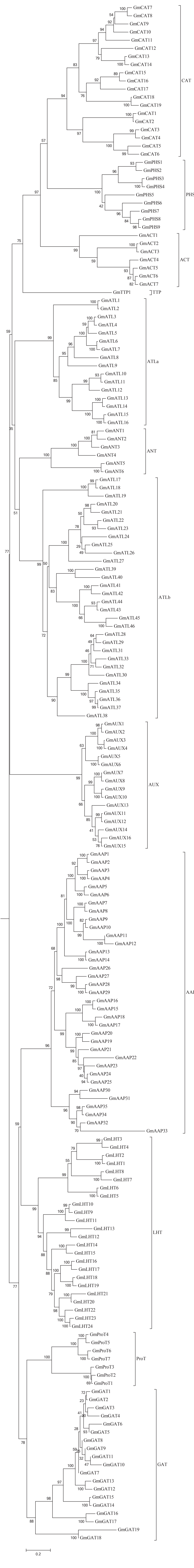

Supplement: Figure S1 — Phylogenetic tree of the nucleotide sequences of AAT genes in soybean. [file Image1.PDF]

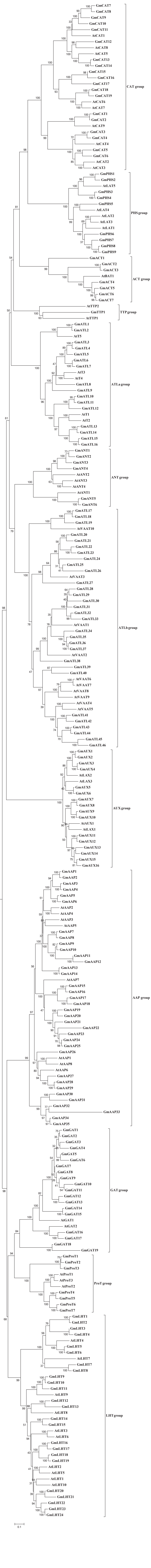

Supplement: Figure S2 — Phylogenetic tree of the nucleotide sequences of AAT genes in soybean and Arabidopsis. [file Image2.PDF]
